# *Prox1* maintains taste bud structure via inhibition of apoptosis

**DOI:** 10.1007/s00441-025-04040-7

**Published:** 2026-02-05

**Authors:** Aya Hagimoto, Eriko Koyanagi-Matsumura, Norihito Oura, Mitsuru Saito, Tatsurou Tanaka, Hirohito Miura

**Affiliations:** 1 Department of Maxillofacial Radiology, Kagoshima University Graduate School of Medical and Dental Sciences, 8-35-1 Sakuragaoka, Kagoshima-Shi, Kagoshima 890-8544 Japan; 2 Department of Oral Physiology, Kagoshima University Graduate School of Medical and Dental Sciences, 8-35-1 Sakuragaoka, Kagoshima-shi, Kagoshima 890-8544 Japan; 3 Department of Oral and Maxillofacial Surgery, Kagoshima University Graduate School of Medical and Dental Sciences, 8-35-1 Sakuragaoka, Kagoshima-shi, Kagoshima 890-8544 Japan

**Keywords:** *Prox1*, Taste bud, Cell turnover, Apoptosis, Cell lifespan

## Abstract

Taste buds are maintained by continuous cell renewal, receiving a steady influx of postmitotic cells from the surrounding epithelial region. Within taste buds, Type I, II, and III cells continuously differentiate from Type IV postmitotic precursor cells and are removed via apoptosis. These processes are likely governed by various transcription factors. Among the transcription factors expressed in taste buds, *Prox1*, a homeobox transcription factor, is the only factor expressed in all taste bud cells including precursor cells. However, its role in taste buds remains unclear. Here, we investigate the function of *Prox1* in taste bud cell turnover using conditional knockout (cKO) mice. In *Prox1* cKO mice, all Type I, II, and III cells were significantly reduced, resulting in approximately half the total cell number per taste bud compared to wild-type mice, while Type IV cell numbers remained comparable. Apoptosis of taste bud cells nearly doubled, leading to a shortened lifespan of taste bud cells. EdU pulse-labeling experiments revealed a biphasic decline in EdU(+) taste bud cells in *Prox1* cKO mice, indicating that *Prox1* knockout increases the fraction of cells that die shortly after differentiating into taste bud cells. The surviving cells still exhibited a shorter lifespan than that of wild-type mice. We also observed previously unreported structural alterations within taste buds caused by enhanced apoptosis using whole-mount analysis. These results demonstrate that *Prox1* contributes to the maintenance of taste bud structure by regulating the lifespan of taste bud cells, highlighting its essential role in taste bud homeostasis.

**Supplementary Information:**

The online version contains supplementary material available at 10.1007/s00441-025-04040-7.

## Introduction

Gustation is an essential sensory function that enables animals to select foods appropriate for their survival. Among the five basic tastes, sweet, umami, and salty tastes facilitate the selection of beneficial nutrients to maintain homeostasis, whereas sour and bitter tastes play a critical role in avoiding spoiled or poisonous items, thereby protecting animals from potential harm.

Taste buds, the sensory end organ for gustation, are located in three types of taste papillae on the tongue (fungiform (FF), circumvallate (CV), and foliate (FL)) and on the soft palate (SP) in the oral cavity. Each taste bud is composed of 50–100 cells and is maintained by continuous cell renewal (Chaudhari and Roper 2010). Taste bud cells are classified into four cell types: Type I, II, and III cells, which are functionally mature, and immature Type IV cells (Finger and Barlow 2021). Stem cells of taste buds reside within the basal layer of the epithelium expressing keratin 5 and 14 (K5 and K14) around taste buds (Okubo et al. 2009). These stem cells are bipotential, giving rise to both taste bud cells and the surrounding epithelium.

Taste bud cells are postmitotic. Cells that complete their terminal division enter the taste buds and begin to express *Sonic hedgehog* (*Shh*), an evolutionarily conserved signaling molecule that regulates cell proliferation and differentiation (Miura et al. 2001, 2006). *Shh*(+) cells differentiate into mature cell types, losing *Shh* expression (Miura et al. 2014). A recent study demonstrated an average half-life of 11 days for taste bud cells, consistent with the long-standing estimate of a 10- to 14-day lifespan, while also noting distinctive longevities among different cell types (Beidler and Smallman 1965; Farbman 1980; Delay et al. 1986; Perea-Martinez et al. 2013). Taste bud cells that complete their lifespan undergo apoptosis (Zeng and Oakley 1999). It was recently reported that Type I cells may be responsible for eliminating dying cells through phagocytosis (Wilson et al. 2025). Nonetheless, many details regarding cell death within taste buds remain unknown (Lakshmanan et al. 2022).

Understanding the roles of transcription factors is essential for elucidating the molecular mechanisms that maintain the homeostasis of taste bud structure and function. *Pou2f3* was the first transcription factor revealed to control cell type differentiation within taste buds, being responsible for the differentiation of Type II and amiloride-sensitive sodium taste cells (Matsumoto et al. 2011; Ohmoto et al. 2020). For Type III cells, *Ascl1* and *Nkx2-2* were identified as specific transcription factors (Miura et al. 2006). Recent studies demonstrated that these factors drive Type III cell differentiation (Seta et al. 2011; Qin et al. 2021). Although no transcription factors were definitively shown to be specific for Type I cells, candidates for specific transcription factors were proposed based on detailed expression analysis using single-cell RNA sequencing (Vercauteren Drubbel and Beck 2023). All these transcription factors exhibit cell type-specific functions.

In contrast, *Prox1*, a homeobox transcription factor, is at present the only transcription factor expressed in all taste bud cells (Nakayama et al. 2015). *Prox1* expression begins in the basal cells of taste buds simultaneously with *Shh* expression during taste bud cell differentiation. As taste cell differentiation progresses, *Shh* expression disappears, whereas *Prox1* persists throughout the lifespan of taste bud cells (Miura et al. 2006; Nakayama et al. 2015). Given this unique expression pattern, *Prox1* is considered to play an important role in the maintenance and regulation of taste buds.

A wide variety of functions were reported for *Prox1* outside taste buds. During development, *Prox1* plays critical roles in the formation of various tissues, including the lymphatic system, nervous system, liver, and pancreas (Wigle and Oliver 1999; Kim et al. 2010; Westmoreland et al. 2012; Seth et al. 2014; Stergiopoulos et al. 2015). In adulthood, *Prox1* serves several critical functions, including supporting neurogenesis in the hippocampus, maintaining the intestinal stem cell niche, and promoting liver regeneration (Stergiopoulos et al. 2015; Goto et al. 2017; Middelhoff et al. 2020). It was shown that *Prox1* can serve as either an oncogene or a tumor suppressor gene during tumorigenesis depending on the context (Rodrigues et al. 2014; Ntikoudi et al. 2022; Lim et al. 2025). However, the specific function of *Prox1* in taste buds remains to be elucidated.

In this study, we analyzed the impact of *Prox1* knockout on taste buds and demonstrated that *Prox1* maintains the lifespan of taste bud cells and preserves taste bud size. In addition, we discovered unreported ovoid spaces within taste buds, which we termed dark voids. The dark voids may reflect structural changes within the taste buds caused by elevated cell death in *Prox1* knockout mice.

## Materials and methods

### Experimental animals

Adult mice older than 8 weeks of age and 2.5-day-old mice were used in the experiments. For the analysis of cell lifespan and cell death, mice aged 12–23 weeks were used. Three or four mice per experimental group were used, and the number of mice used is shown in each figure legend. Both male and female mice were used in the experiments. Animals were housed at 23 °C under a 12:12-h light/dark cycle with free access to standard chow and water. All experimental procedures were approved by the Institutional Animal Care and Use Committee and conducted at Kagoshima University.

Because the conventional knockout of *Prox1* causes embryonic lethality (Wigle and Oliver 1999), we adopted a conditional knockout approach. *Prox1* floxed mice, in which exon 2 (containing the homeodomain) is flanked by two loxP sequences (Goto et al. 2017), were generously provided by Dr. Hajime Kubo of Kyoto University. Cre recombinase expression was driven by the *Keratin5* promoter using *Keratin5-Cre* mice (STOCK-*Tg(K5-Cre)Jt*) (Tarutani et al. 1997; Mikami et al. 2024; Horie et al. 2025) obtained from CARD R-BASE, Kumamoto University, as taste bud cells are derived from stem cells expressing keratin 5 (Okubo et al. 2009). Both mouse strains had a C57BL/6J genetic background.

We used the following genotypes: *K5-Cre;Prox1*^*fl/fl*^, *K5-Cre;Prox1*^*fl/*+^, *K5-Cre;Prox1*^+*/*+^, and C57BL/6J. C57BL/6J mice were purchased from The Jackson Laboratory Japan (Yokohama, Japan).

We confirmed that PROX1 expression is comparable between C57BL/6J and *Prox1*^*fl/fl*^ mice by immunohistochemistry (Supplementary Fig. 1).

### Tissue preparations

The mice were euthanized by cervical dislocation, and the tissues were harvested.

For immunohistochemistry on tissue sections, the tongue and soft palate were harvested, embedded immediately in Tissue-Tek® O.C.T. Compound (Sakura Finetek, Tokyo, Japan), and frozen without prior fixation. The tissues were stored at − 80 °C, and frozen tissues were sectioned to a thickness of 5 µm. The sections were stored at − 80 °C until use and fixed with 4% paraformaldehyde (PFA) in phosphate-buffered saline (PBS) at room temperature for 10 min at the start of immunohistochemistry. For 2.5-day-old mice, tissues were fixed in 4% PFA in PBS at 4 °C for 3 h before embedding in O.C.T. compound.

For whole-mount immunohistochemistry, taste epithelia from the SP, FF, and CV were obtained following enzymatic treatment, as described previously (Koyanagi-Matsumura et al. 2021). Briefly, Ringer’s solution containing 2.5 mg/ml collagenase type IV (Worthington Biochemical, Lakewood, NJ) and 2 mg/ml elastase (Worthington Biochemical) was injected under the epithelium, and the tissues were incubated at 37 °C for 30 min. The epithelium was then peeled off using forceps in PBS, fixed with 4% PFA in PBS, and processed for immunohistochemistry. The fixation conditions of temperature and time are summarized in Supplementary Table 1.

### Immunohistochemistry

For immunohistochemistry on slices, antigen retrieval was performed by heating the sections in 0.1 M citrate buffer (pH 6.0) at 100 °C for 15 min. After rinsing three times for 5 min each with TBST (Tris-buffered saline (TBS) containing 0.05% Tween 20), the sections were incubated in TBSB1 (TBS containing 5% normal donkey serum and 0.3% Triton X-100) for 1 h at room temperature to block non-specific staining. The tissues were then incubated in TBSB2 (TBS containing 2.5% normal donkey serum and 0.1% Triton X-100) containing primary antibodies (Supplementary Table 2) overnight at 4 °C. Following three washes with TBST for 5 min each, the sections were incubated overnight at 4 °C with secondary antibodies (Supplementary Table 3) diluted in TBSB2. After washing three times with TBST for 5 min each, the sections were rinsed with TE (10 mM Tris–HCl (pH 8.0), 1 mM EDTA) and coverslipped with ProLong Glass Antifade Mountant with NucBlue (Thermo Fisher Scientific, MA, USA). For sections from 2.5-day-old mice, TO-PRO-3 (Invitrogen, Thermo Fisher Scientific, MA, USA) was applied at room temperature for 30 min before washing with TE and coverslipped with ProLong Glass Antifade Mountant (Thermo Fisher Scientific).

Whole-mount immunohistochemistry was performed as previously described, with some modifications (Koyanagi-Matsumura et al. 2021). The peeled epithelium was incubated in 10 mM citrate (pH 6.0) at 105 °C for 1 min (to detect cleaved caspase-3) or 3 min (to detect SHH) using an electric pressure cooker (EL-MB30, Zojirushi Corp., Osaka, Japan) for antigen retrieval. The tissues were washed three times with TBST for 15 min each. The antigen retrieval step was omitted for the detection of other antigens. The tissues were incubated in TBSB1 for 1 h at room temperature and incubated with primary antibodies diluted in TBSB2 overnight at 4 °C (Supplementary Table 2). The tissues were washed three times with TBST for 15 min each and incubated with secondary antibodies in TBSB2 containing Hoechst 33342 (Invitrogen, Thermo Fisher Scientific, MA, USA) overnight at 4 °C (Supplementary Table 3). The tissues were washed three times with TBST for 15 min each. Finally, the tissues were rinsed with TE, mounted, and coverslipped with RapiClear® 1.49 (SUNJin Lab, Hsinchu, Taiwan).

### Image processing

Immunofluorescence was imaged using a Leica TCS SP8 confocal microscope with a 40 ×/1.3 or 63 ×/1.4 oil immersion lens. For whole-mount immunohistochemistry, a series of optical sections were acquired at 0.5–1.0 µm intervals from the bottom to the apical portion of the taste buds. Three-dimensional images were reconstructed from the z-stack and resliced using Imaris software version 10.0.2 (Bitplane AG, Zürich, Switzerland).

To analyze the distribution and number of taste buds in the FF and SP, fluorescent signals for KCNQ1 were detected using a Keyence BZ-X700 all-in-one fluorescence microscope with a 20 ×/0.75 objective lens.

Brightness and contrast were adjusted using Imaris and/or Adobe Photoshop (Adobe Systems, San Jose, CA, USA).

### Statistical analysis

Statistical software KaleidaGraph version 5.0.6 (Synergy Software, Reading, PA, USA) and R version 4.5.0 (https://www.r-project.org) were used for the statistical analysis.

Before comparing the two groups using the *t*-test, we tested for homogeneity of variances using the *F*-test and confirmed that no significant variance heterogeneity was present across all datasets in this study. Therefore, Student’s *t*-test was used for all two-group comparisons. For categorical data, comparisons between groups were performed using the chi-squared test. For multiple group comparisons, ANOVA was performed, followed by post hoc testing using the Tukey’s HSD correction. The threshold for statistical significance was set at *α* = 0.05.

### Quantification of taste bud cells: numbers of total cells and Type II/III cells per taste bud

To quantify the total number of cells per taste bud in the CV, cell segmentation was performed using Cellpose version 3.0.1 (Stringer et al. 2021; Chan Zuckerberg Biohub, https://cellpose.org), a deep learning-based segmentation tool. The cyto3 generalist model was used for segmentation, with the diameter parameter set to 30 and the cell probability threshold set to 3, based on KCNQ1 signals detected by whole-mount immunohistochemistry. The default settings were used for all other parameters.

Segmentation masks generated by Cellpose were imported into Imaris software as surface objects. Masks not derived from taste bud cells, most of which originated from the ductal KCNQ1(+) cells of von Ebner’s gland, were removed. Since the import from Cellpose to Imaris was image-based (pixel/dot data) rather than coordinate or direct segmentation data transfer, small gaps between adjacent segments appeared as small artifact surfaces during the import process. To exclude these artifacts, an empirically determined volume threshold of 100 µm^3^ was applied, and surface objects smaller than this threshold were removed from the analysis. The total number of cells per taste bud was then determined by counting the remaining surface objects.

To quantify the number of Type II and III cells, IP3R3(+) and CA4(+) cells in the SP, FF, and CV were detected by whole-mount immunohistochemistry. IP3R3(+) and CA4(+) cells in each taste bud were quantified using the cell counter plugin of Fiji/ImageJ (National Institutes of Health, Maryland, USA), as described previously (Koyanagi-Matsumura et al. 2021).

### EdU administration and quantification of EdU(+) cells

EdU solution (50 mg/kg body weight) was administered via a single intraperitoneal injection at 10:00 a.m. The CV epithelium was collected and used for the following analyses (at the respective time): 1) analysis of proliferating cells (2 h after injection) and 2) analysis of taste bud cell lifespan (at 10:00 a.m. on days 4, 8, 12, 16, 20, and 28 post-injection). The peeled CV epithelium was processed for whole-mount immunohistochemistry to detect KCNQ1, and then EdU was detected using Click-iT® Plus EdU Imaging Kits (Thermo Fisher Scientific) according to the manufacturer’s protocol.

1. Analysis of proliferating cells

EdU(+) cells were counted in 200 µm × 100 µm rectangular regions of the CV epithelium, which were selected to include a similar number of taste buds among the counting samples using Imaris software. Two regions were selected from each mouse and analyzed. To detect individual EdU(+) nuclei, the Spot tool in Imaris was used with a diameter threshold of 4.4 µm. This value was empirically determined to reliably identify one spot per EdU-labeled nucleus. Nuclei on one long and one short edge of each counting region analyzed were systematically excluded to avoid double counting. The number of proliferating cells was expressed as the number of spots detected per 1000 µm^2^.

2) Analysis of taste bud cell lifespan

Two trench walls of the CV from each mouse were analyzed. The number of EdU(+) cells within taste buds and the number of taste buds were counted at each time point after EdU injection, and the mean number of EdU(+) cells per taste bud was calculated. Temporal changes in the number of EdU(+) cells per taste bud were plotted. For wild-type mice, a one-phase exponential decay curve was fitted starting from day 12 post-injection, when the number of EdU(+) cells began to decline. For *Prox1* cKO mice, two exponential decay curves were fitted: one for days 4–12 post-injection and another for days 12–28. The decay curve was modeled using the equation *y* = *m1* × e^−*m2x*^, where *m1* represents the estimated initial value at *x* = 0 and *m2* represents the decay constant, using KaleidaGraph software. The half-life and lifespan of EdU(+) cells were calculated based on the decay constants. The lifespan of taste bud cells was calculated by adding the plateau period to the lifespan calculated based on the decay curve.

### Analysis of cell death in taste buds

Two trench walls of the CV from each mouse were analyzed. NTPDase2 and cleaved caspase-3 (CC3) were detected using whole-mount immunostaining.

To quantify apoptotic cell death within the taste buds, NTPDase2 signals were initially reconstructed as three-dimensional surface objects using Imaris software. CC3 signals included in NTPDase2-defined surface objects were isolated using mask-based signal extraction. Subsequently, CC3 signals were reconstructed as surface objects. The total volumes of both NTPDase2 and CC3 surface objects were calculated. The ratio (%) of the total volume of CC3 surface objects to that of NTPDase2 surface objects was used as an indicator of cell death per taste bud.

### Summary of statistical data

All P values in every statistical analysis in this study are available in the Supplementary Excel file.

## Results

### Taste buds with reduced size persist after *Prox1 *deletion

We adopted a conditional knockout approach to investigate the function of *Prox1* in taste buds because the conventional knockout of *Prox1* results in embryonic lethality (Wigle and Oliver 1999). *Keratin5-Cre* was used to conditionally knock out the *Prox1* gene in stem cells for taste buds using the Cre/loxP system.

**Fig. 1:**
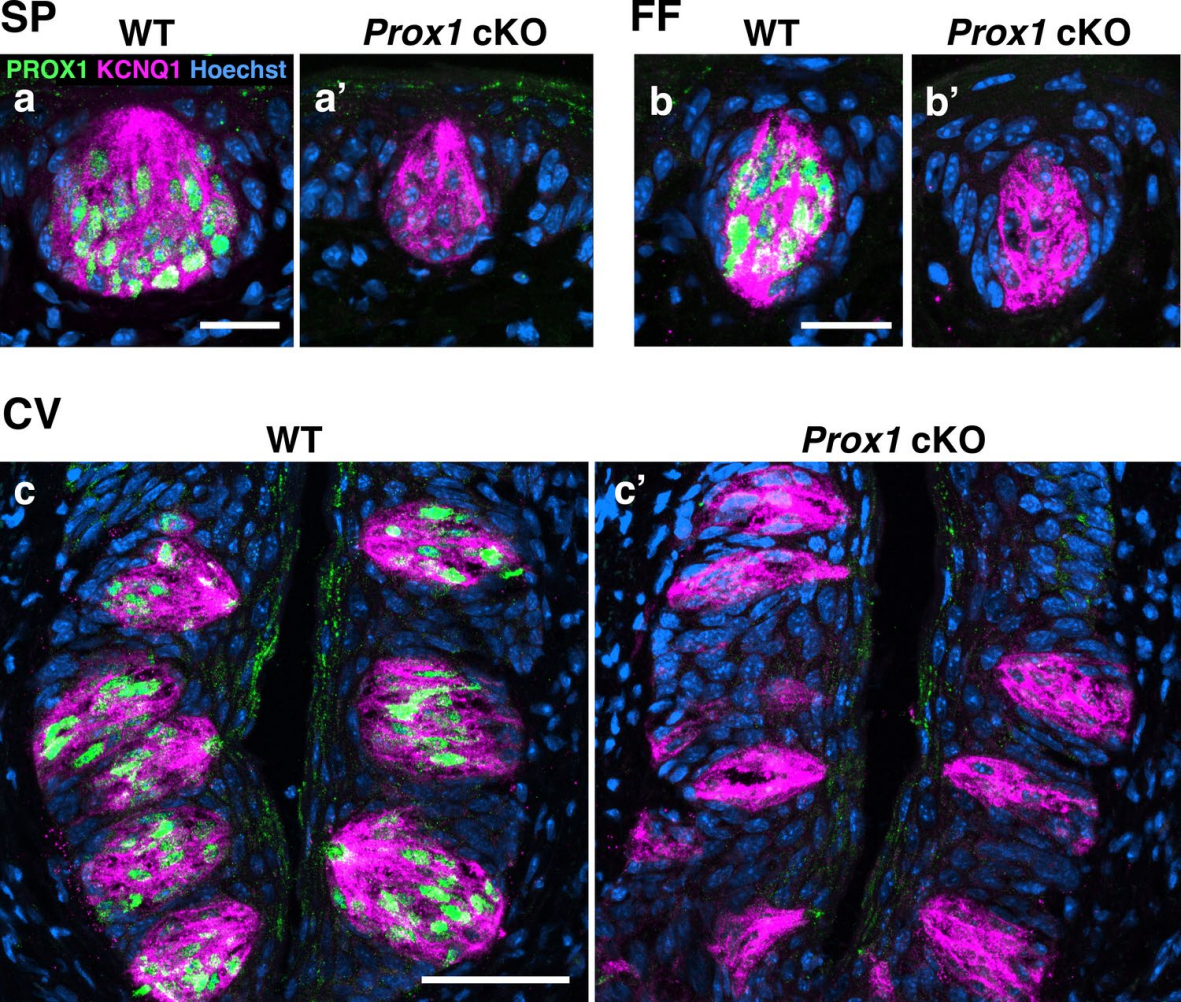
Immunohistochemistry on tissue sections showing PROX1 (green) and KCNQ1 (magenta) in the soft palate (SP), fungiform papillae (FF), and circumvallate papillae (CV). Taste buds were detected by KCNQ1 (magenta) immunostaining in wild-type mice (**a**, **b**, **c**) and *K5-Cre;Prox1*^*fl/*^^*fl*^ (*Prox1* cKO) mice (**a’**, **b’**, **c’**). The expression of PROX1 (green) was abolished in *Prox1* cKO mice, whereas taste buds remained present but were reduced in size. Scale bars: 20 µm (SP and FF) and 50 µm (CV)

To confirm whether *Keratin5-Cre* (*K5-Cre*) effectively induced *Prox1* gene knockout in taste buds, we performed immunohistochemistry for PROX1 in combination with the taste cell marker KCNQ1 on tissue sections in the soft palate (SP), fungiform papillae (FF), and circumvallate papillae (CV). In *K5-Cre;Prox1*^*fl/fl*^ (*Prox1* cKO) mice, while taste buds were clearly detected by KCNQ1 expression in all regions, PROX1 expression was absent in KCNQ1(+) cells, except for a few residual cells, indicating that *Prox1* was successfully knocked out in the taste buds (Fig. 1). Although taste buds persisted in *Prox1* cKO mice, their size seemed to be reduced compared to that in wild-type controls.

Keratin 5 and keratin 14 are known to be co-expressed as a pair specifically in basal cells of the epithelium containing taste bud stem cells. A recent study reported that cells that give rise to taste buds are keratin 14(−) at the embryonic stage in the FF and shift to keratin 14(+) postnatally (Kramer et al. 2019; Golden et al. 2021). This suggests that *K5-Cre* does not efficiently induce *Prox1* cKO in taste buds during the early postnatal stage. To assess this possibility, we examined the taste buds in the FF and SP of 2.5-day-old *K5-Cre;Prox1*^*fl/fl*^ mice (Supplementary Fig. 2). In the FF, most taste buds contained multiple PROX1(+) cells, indicating that *Prox1* was not knocked out at this developmental stage. Although most taste buds in the SP lacked PROX1(+) cells, taste buds containing a few PROX1(+) cells were occasionally observed. These results suggest that *Prox1* deletion may start in early postnatal stages but is not completed until adulthood.

Therefore, we used *K5-Cre;Prox1*^*fl/fl*^ mice older than 8 weeks of age as *Prox1* cKO mice, at which point taste bud stem cells had shifted to keratin 5(+).

### Distribution and number of taste buds are not altered in *Prox1 *cKO mice

**Fig. 2:**
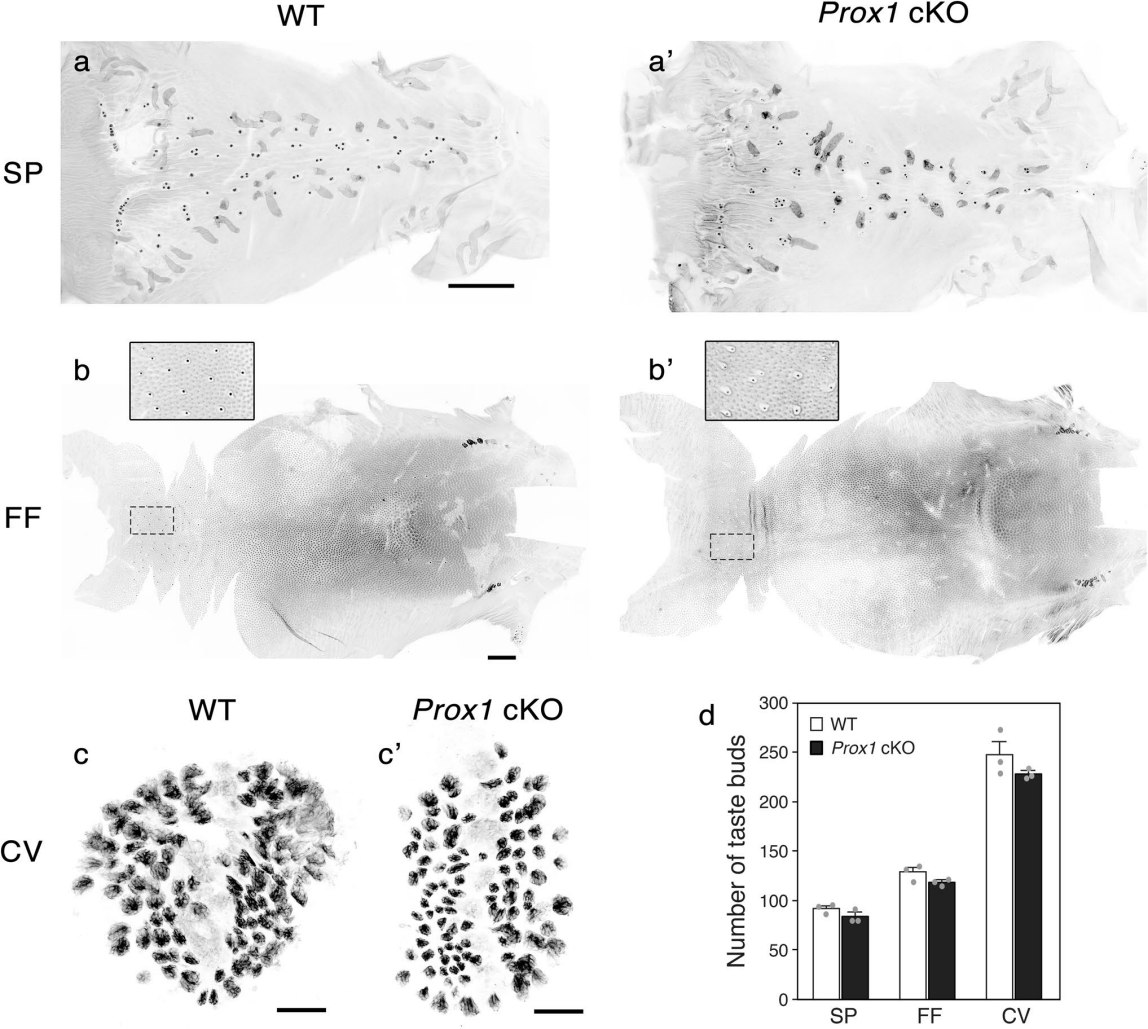
Taste bud number and distribution in the SP, dorsal tongue (FF), and CV. Flat-mounts of peeled epithelium of the SP (**a**, **a’**), FF (**b**, **b’**), and CV (**c**, **c’**). Taste buds were detected by whole-mount immunostaining for KCNQ1. **a–b’** The anterior side is oriented to the left in the SP and FF. **b**, **b’** High-magnification images were superimposed for the areas outlined by dashed lines in the FF. **c**, **c’** The CV epithelium was shown with the bottom of the trench positioned centrally and the trench walls on both sides. No apparent differences in taste bud distribution were observed between wild-type and *Prox1* cKO mice in any of the regions. Scale bar: 1 mm (SP and FF) and 100 µm (CV). **d** Number of taste buds in the SP, FF, and CV (mean ± SE, *n* = 3 per group). The individual data points are represented by gray dots. No significant differences were observed between wild-type and *Prox1* cKO mice in any of the regions (Student’s *t*-test)

Having confirmed the effective deletion of *Prox1*, we next explored its impact on the spatial arrangement and quantity of taste buds in each region. The distribution and number of taste buds were analyzed using KCNQ1 whole-mount immunohistochemistry of peeled epithelium from the tongue and palate (Fig. 2). No apparent differences were observed in taste bud distribution between wild-type and *Prox1* cKO mice in the SP, FF, and CV. No significant difference in the number of taste buds was observed between wild-type and *Prox1* cKO mice in any of the regions (SP, *p* = 0.170; FF, *p* = 0.168; CV, *p* = 0.277; Student’s *t*-test).

### Type I–III cells differentiate but are reduced in *Prox1 *cKO mice

**Fig. 3:**
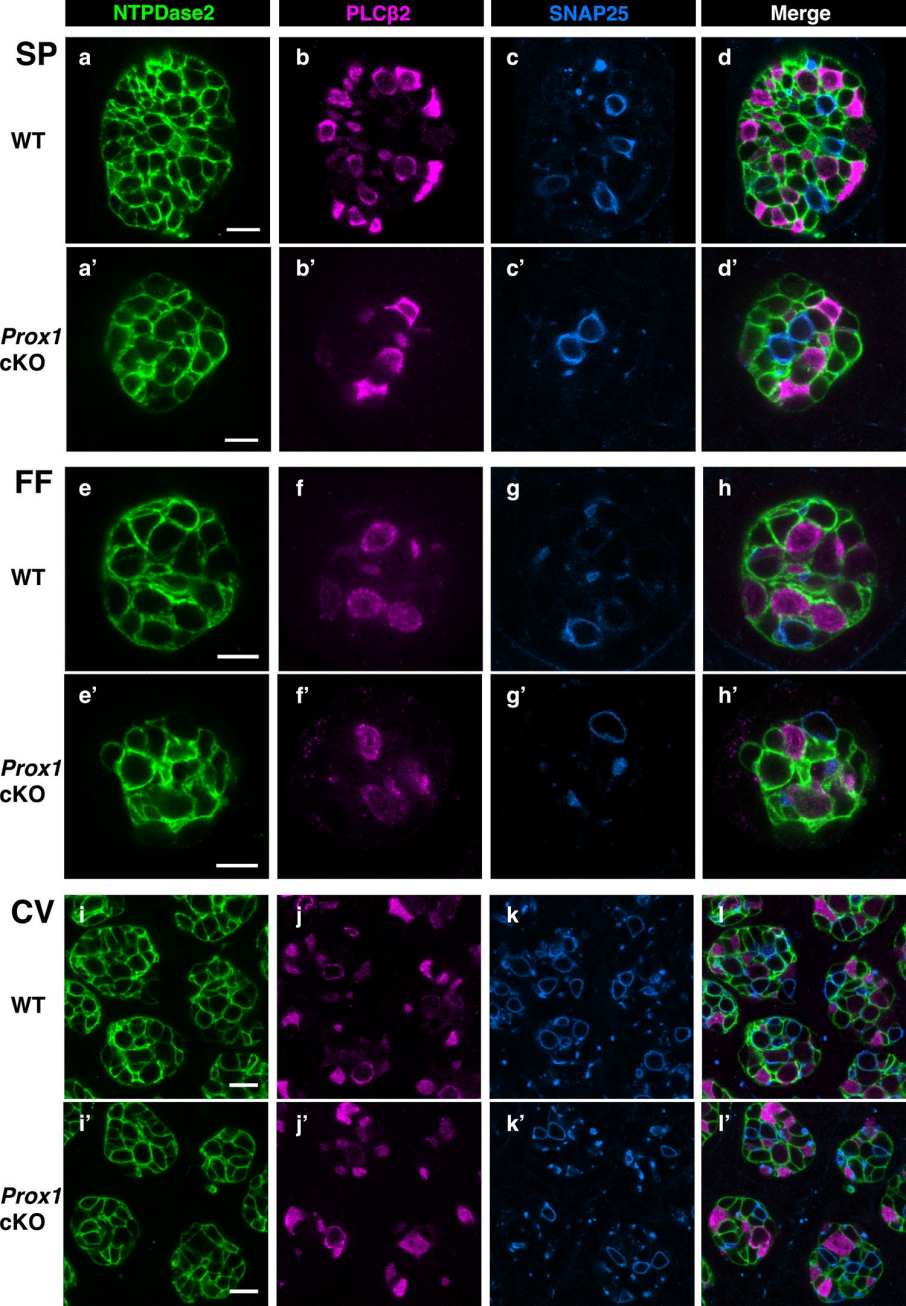
Triple-color whole-mount immunohistochemistry for Type I–III cell markers in example taste buds on the SP, FF, and CV. Optical sections showing the expression of NTPDase2 (green), PLCβ2 (magenta), and SNAP25 (blue) in the SP (**a**–**d**), FF (**e**–**h**), and CV (**i**–**l**) of wild-type mice, and in the SP (**a’**–**d’**), FF (**e’**–**h’**), and CV (**i’**–**l’**) of *Prox1* cKO mice. Scale bar: 10 µm

After confirming that the distribution and number of taste cells were not significantly different between wild-type and *Prox1* cKO mice, we investigated the impact of *Prox1* knockout on cell differentiation within the taste buds. To determine whether all mature cell types exist in *Prox1* cKO mice, we performed whole-mount immunohistochemistry using markers for Type I (NTPDase2), Type II (PLCβ2), and Type III (SNAP25) cells in the SP, FF, and CV. To simultaneously visualize all three cell types within individual taste buds, we employed triple-color immunostaining, which allowed us to assess the cellular composition of each taste bud in detail. All mature cell type markers were detected in both wild-type and *Prox1* cKO mice in all three regions, and the proportions of each cell type appeared similar between the two mouse groups. Among the three cell types, Type I cells were the most abundant, followed by Type II cells, and Type III cells were the least abundant (Fig. 3, Supplementary Fig. 3). However, the numbers of Type I, II, and III cells appeared to be reduced in *Prox1* cKO mice in each region.

Subsequently, we quantified the number of mature cells per taste bud using whole-mount immunohistochemistry, which allowed us to determine the precise number of each cell type in individual taste buds (Koyanagi-Matsumura et al. 2021). Type I cells have a complex morphology with thin cell processes wrapping neighboring cells, and it is difficult to accurately quantify the cell numbers (Miura et al. 2014). Therefore, we analyzed Type II and III cells using IP3R3 and CA4 as cell-type markers, respectively. To ensure that the expression of Cre recombinase itself in keratin5(+) cells does not affect the cell number within taste buds, we included *K5-Cre*;*Prox1*^+*/*+^ mice as an additional control group.

**Fig. 4:**
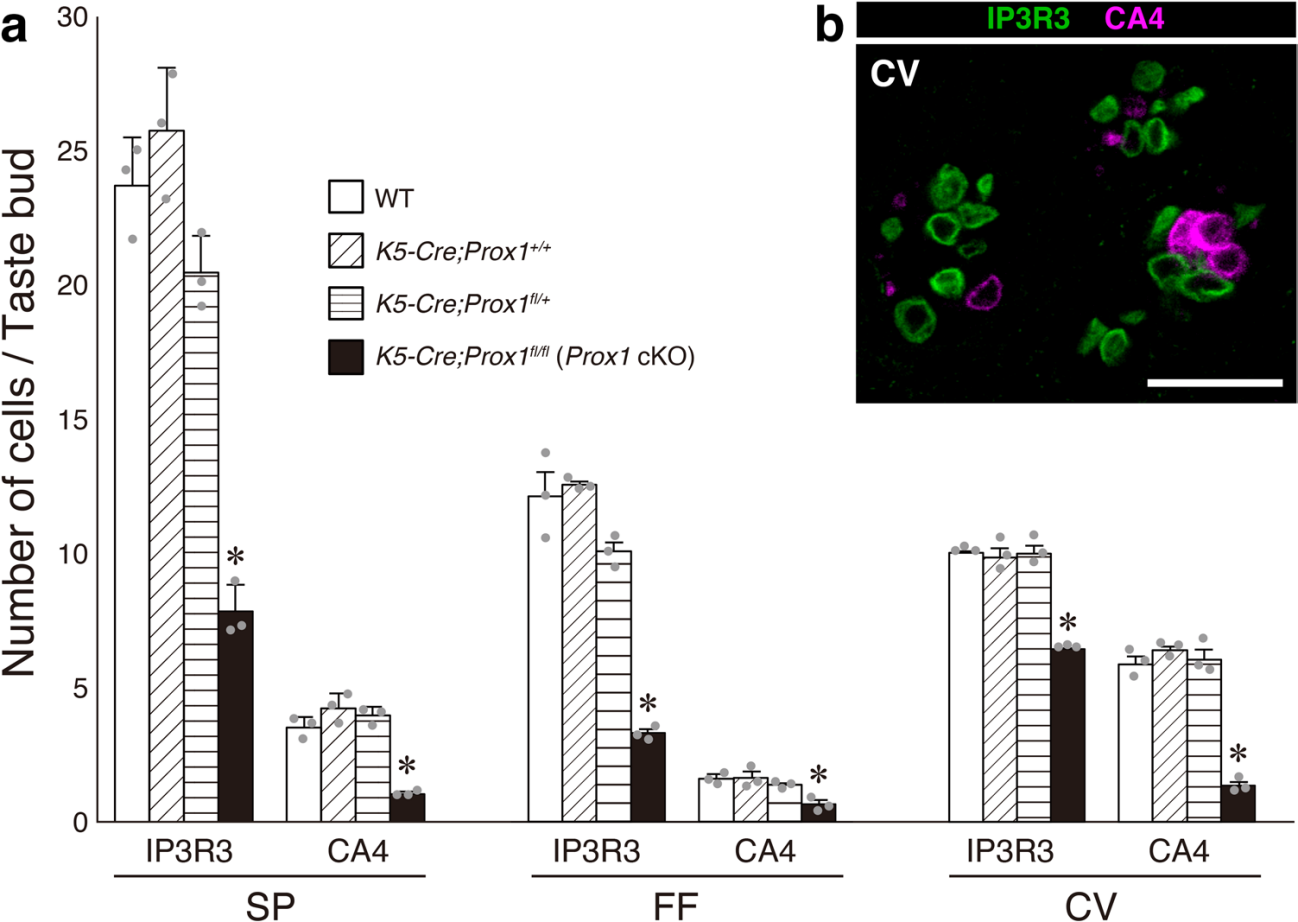
Number of Type II and III cells per taste bud in the SP, FF, and CV analyzed by whole-mount immunohistochemistry. **a** The number of IP3R3(+) and CA4(+) cells per taste bud in each region is shown for wild-type, *K5-Cre;Prox1*^+*/*+^, *K5-Cre;Prox1*^*fl/*+^, and *K5-Cre;Prox1*^*fl/fl*^ (*Prox1* cKO) mice (mean ± SE, n = 3 per group). The individual data points are represented by gray dots. Asterisks indicate significant differences compared to wild-type mice in each region (*p* < 0.05; one-way ANOVA, post hoc Tukey’s HSD test). The number of IP3R3(+) and CA4(+) cells was significantly decreased in all regions in *Prox1* cKO mice, and there was no significant difference between wild-type and *K5-Cre;Prox1*^+*/*+^. **b** An example optical section of the CV in *Prox1* cKO mice is superimposed on the upper right side of the graph (a) to show the quality of immunostaining used for the quantification of IP3R3(+) (green) and CA4(+) (magenta) cells. Scale bar: 30 µm

In *Prox1* cKO mice, the number of IP3R3(+) and CA4(+) cells per taste bud were significantly decreased compared to those in wild-type mice in all three regions (the SP, FF, and CV) (one-way ANOVA, post hoc Tukey’s HSD test) (Fig. 4). In contrast, in *K5-Cre;Prox1*^+*/*+^ mice, no significant differences were observed in any cell type in any region, and the number of IP3R3(+) and CA4(+) cells was comparable to that in wild-type (C57) mice. These results demonstrate that the expression of Cre recombinase in keratin5(+) cells alone does not affect taste bud morphology or cell numbers. Furthermore, Cre recombinase did not affect taste bud distribution, since it was not significantly different between wild-type and *Prox1* cKO mice (Fig. 2). Therefore, we concluded that wild-type mice were appropriate as the control group for subsequent analyses of *Prox1* cKO mice. Collectively, these observations indicate that the reduction in Type I–III cell numbers is attributable to the loss of PROX1 expression.

### *Prox1* cKO reduces taste bud cell number by half and induces dark void formation

**Fig. 5:**
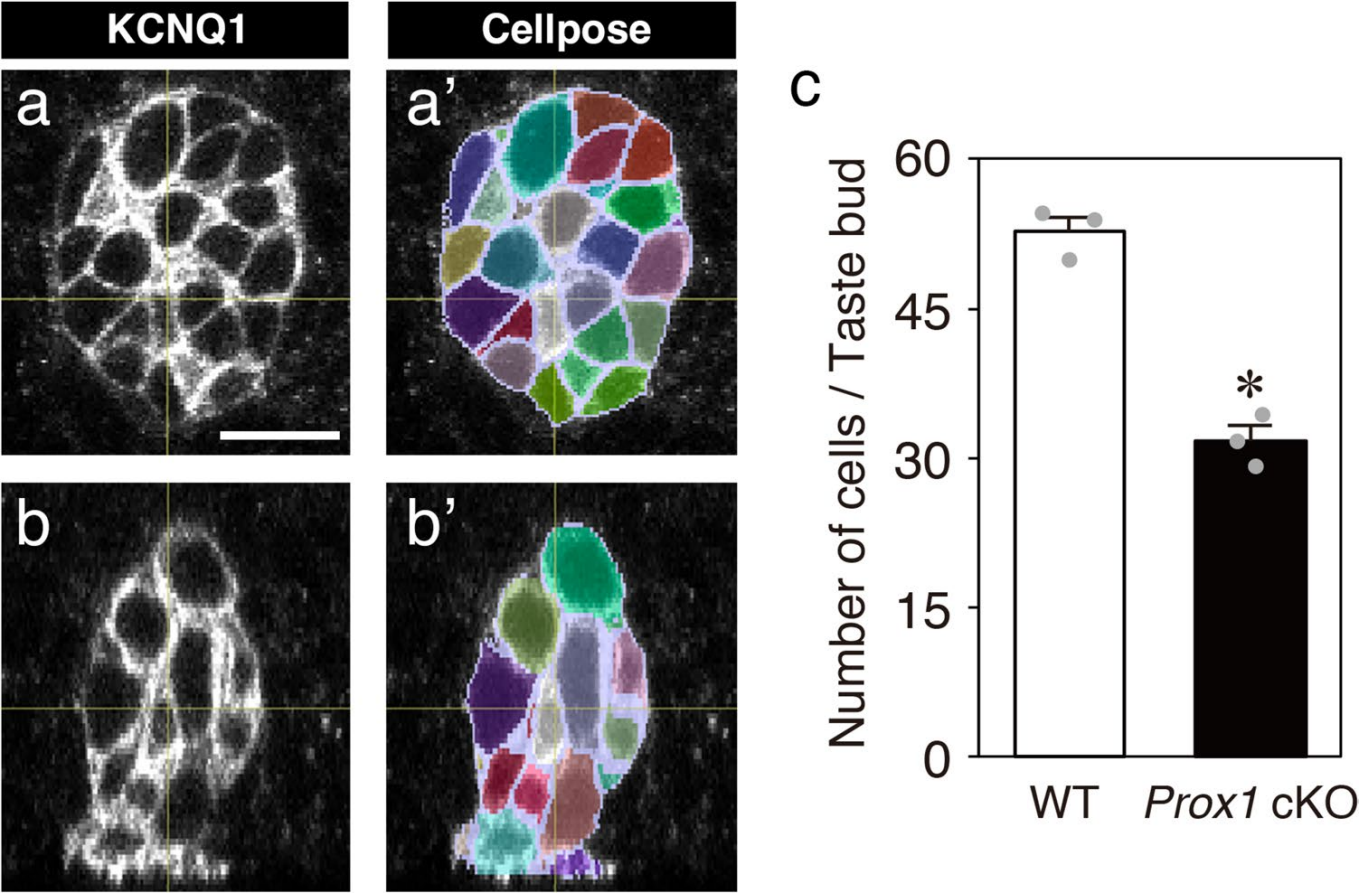
Total number of cells per taste bud in the CV analyzed by whole-mount immunohistochemistry. Transverse (**a**) and longitudinal (**b**) optical sections are shown for an example taste bud of *Prox1* cKO mice obtained from whole-mount immunohistochemistry for KCNQ1. **a’**, **b’** Cellpose-generated individual cell segmentation masks are overlaid on the KCNQ1-immunostainings (**a**, **b**). Scale bar: 10 µm. Individual cell segmentations were generated based on KCNQ1 immunostaining signals using the image analysis software Cellpose, followed by quantification of the taste bud cell numbers. **c** Number of cells per taste bud (mean ± SE, *n* = 3 per group). The individual data points are represented by gray dots. The asterisk indicates a statistically significant difference from that in wild-type mice (*p* < 0.05; Student’s *t*-test). The number of taste bud cells in *Prox1* cKO mice was approximately half that in wild-type mice

Next, to evaluate the impact of the reduction in mature cells on taste bud size, we quantified the total number of cells per taste bud in the CV using KCNQ1 whole-mount immunohistochemistry (Fig. 5). The average cell number per taste bud in *Prox1* cKO mice (31.7 ± 1.5 [mean ± SE]) was approximately half of that in wild-type mice (52.7 ± 1.4), and this difference was statistically significant (*n* = 3 per group, *p* < 0.001, Student’s *t*-test).

**Fig. 6:**
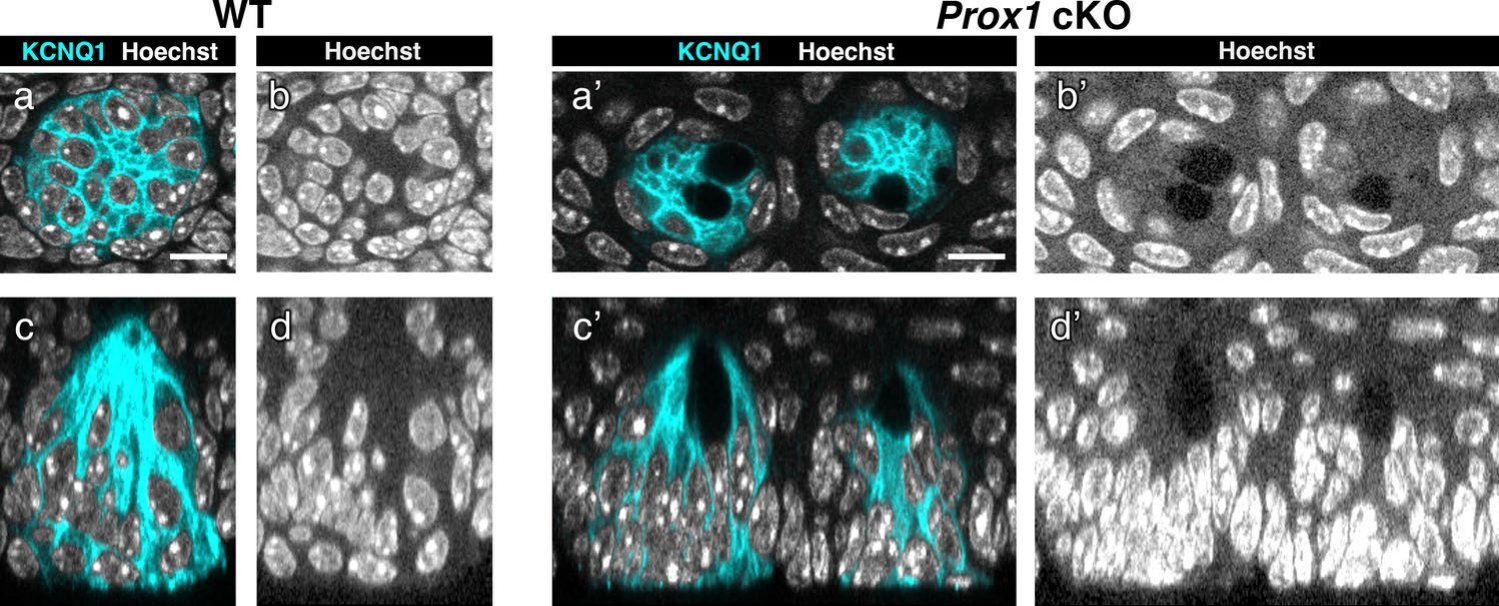
Dark voids in the CV taste buds of *Prox1* cKO mice observed by whole-mount immunohistochemistry. Taste buds were detected by KCNQ1 (cyan). Transverse sections of the taste buds in wild-type mice (**a**, **b**) and *Prox1* cKO mice (**a’**, **b’**). **b**, **b’** Hoechst signals (white) are shown with adjusted gamma values to enhance the visibility of dark voids. In *Prox1* cKO mice, dark voids with no background signals of nuclear staining were apparent in the taste buds. The taste buds shown in (**c**, **d)** and (**c’**, **d’**) are longitudinal sections of the taste buds shown in (**a**, **b**) and (**a’**, **b’**), respectively. The dark voids are located within the taste buds and do not open into the oral cavity. Scale bar: 20 µm. The taste buds in *Prox1* cKO mice included dark voids lacking background signals of nuclear staining (**a’**–**d’**), which were rarely observed in the taste buds of wild-type mice

During this analysis, we unexpectedly encountered unusual ovoid spaces, referred to as dark voids, within the taste buds of *Prox1* cKO mice. The dark voids showed no KCNQ1 signal, no nuclear staining, and even lacked the background signal that normally appears with nuclear staining (Fig. 6; Supplementary Movie). The dark voids are located within the taste buds and do not open into the oral cavity. They were predominantly located in the upper half of the taste buds, where old and dying taste cells are found (Feng et al. 2014; Wilson et al. 2025). Therefore, we carefully examined wild-type mice and found similar structures, which were rare and smaller than those observed in the *Prox1* cKO mice. Dark voids were detected in 15% (151/1000) of taste buds in *Prox1* cKO mice and 2.2% (27/1218) in wild-type mice. The occurrence of taste buds containing dark voids was significantly higher in *Prox1* cKO mice compared to wild-type mice (Chi-squared test, *p* < 0.001). Although extremely rare, small nuclear fragments resembling apoptotic bodies were observed at the periphery within the dark void: 0.3% (3/1000) of taste buds in *Prox1* cKO mice and 0% (0/1218) in wild-type mice (Supplementary Fig. 4).

These results indicate that *Prox1* cKO not only reduces the number of mature taste bud cells but also increases the occurrence of dark voids in the taste buds.

### Immature cells are not changed or slightly increased in *Prox1* cKO mice

**Fig. 7:**
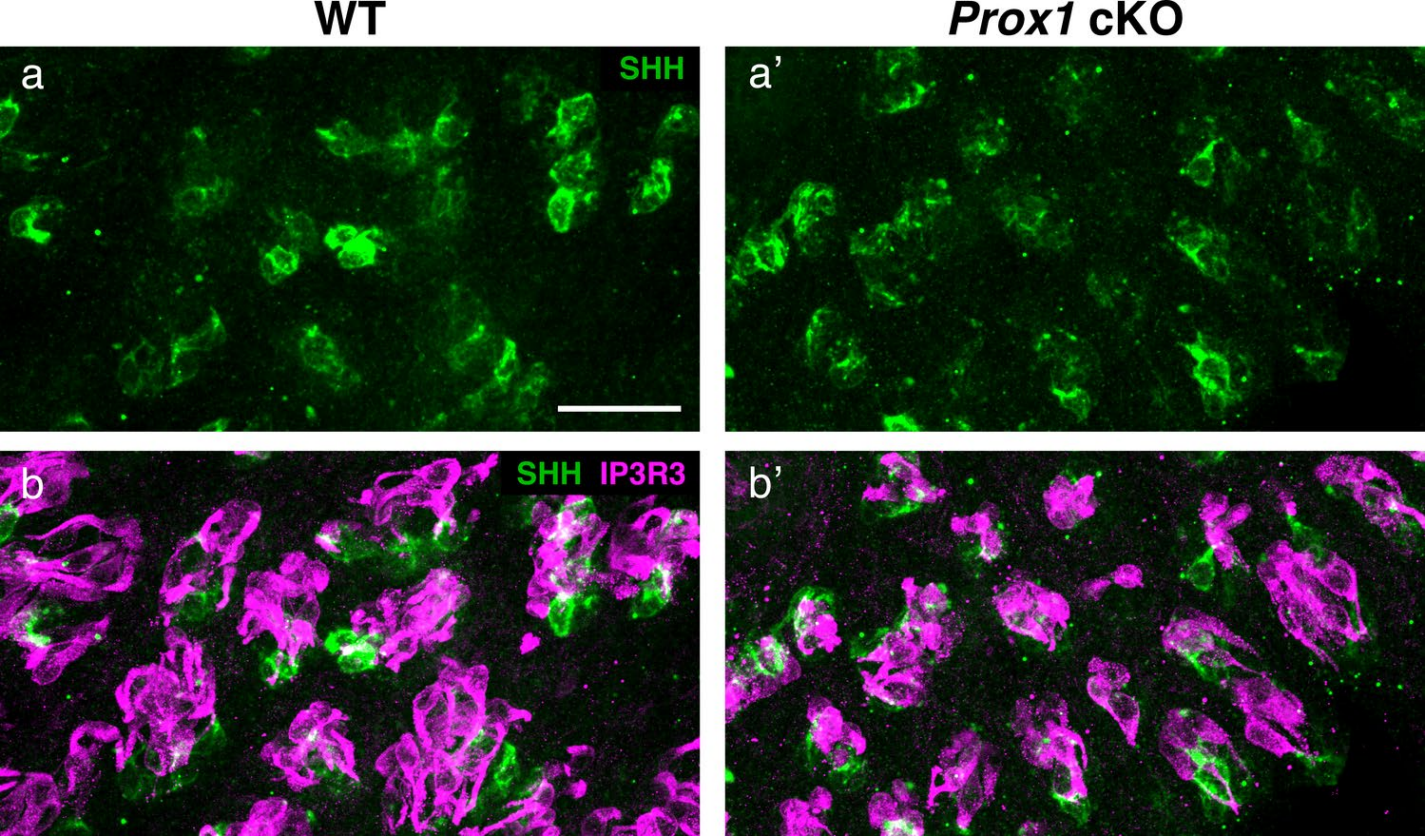
Double-color whole-mount immunohistochemistry of the CV epithelium detecting SHH (green) and IP3R3 (magenta). Three-dimensional projection images of immunofluorescent signals in wild-type (**a**, **b**) and *Prox1* cKO mice (**a’**, **b’**). Images of SHH (**a**, **a’**) and merged images (**b**, **b’**) of SHH and IP3R3 are shown. IP3R3(+) cells indicate the position of the taste buds. SHH signals were readily detected, however, the extremely faint signals spreading in the taste buds made quantification difficult. No apparent differences were observed in SHH immunostaining between wild-type and *Prox1* cKO mice. Scale bar: 30 µm

The significant reduction in taste bud cells observed in *Prox1* cKO mice suggests that alterations in cell turnover dynamics may have occurred. To assess the first step of cell differentiation during taste bud cell turnover, we performed whole-mount immunohistochemistry for SHH. SHH(+) cells are postmitotic transient precursors of Type I–III mature cells in taste buds. In wild-type mice, *Shh*(+) cells emerge from proliferating cells around taste buds approximately 1 day after cell birth, and after about 2 days, they lose *Shh* expression and begin to differentiate into Type I–III cells (Miura et al. 2006, 2014). The number of SHH(+) cells is expected to reflect the number of cells newly supplied to the taste buds from cells surrounding the taste buds. Whole-mount immunohistochemistry readily detected SHH(+) cells in both wild-type and *Prox1* cKO mice, and the number of SHH(+) cells appeared slightly higher in *Prox1* cKO mice than in wild-type mice (Fig. 7). However, it was difficult to profile and accurately quantify SHH(+) cells because the intensity of immunostaining ranged from strong to extremely weak. This variation may be because SHH is a secreted molecule that diffuses into intercellular spaces. Although precise quantification was difficult, SHH immunostaining indicated that, in contrast to the reduction in mature cell types, the number of SHH(+) precursor cells appeared to be maintained after *Prox1* knockout.

### The lifespan of taste bud cells is shortened in *Prox1* cKO mice

To investigate the impact of *Prox1* KO on cell turnover dynamics, we analyzed the lifespan of taste bud cells using EdU pulse-chase labeling in whole-mount preparations.

**Fig. 8:**
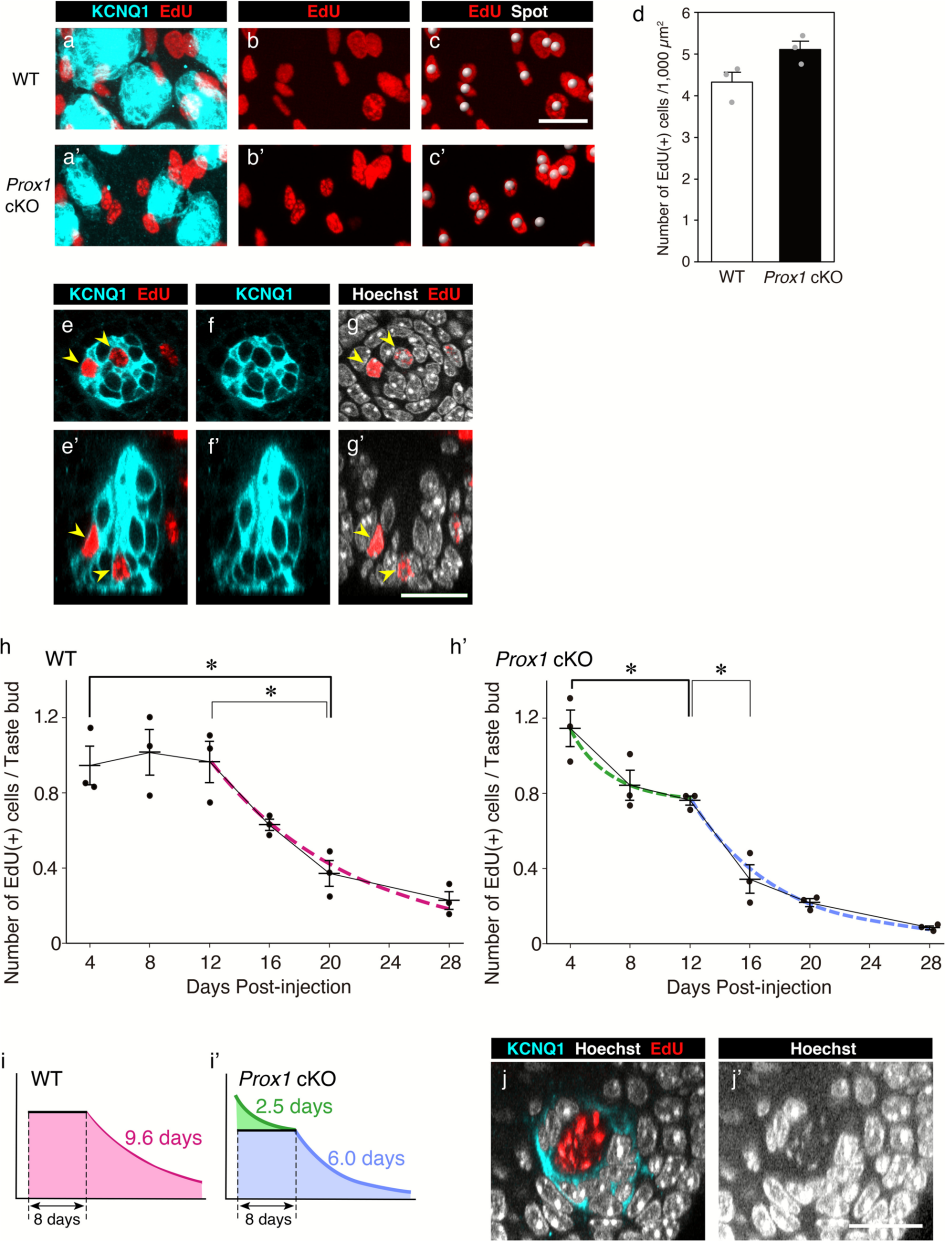
Cell proliferation and lifespan of taste bud cells in the CV. Whole-mount analysis. **a**–**c**, **a’**–**c’** 3D projection images of KCNQ1(+) cells (cyan) and EdU-positive nuclei (red) 2 h after EdU injection in wild-type (**a**–**c**) and *Prox1* cKO mice (**a’**–**c’**). Portions of the analyzed epithelial area (200 µm × 100 µm) are presented. **c**, **c’** White spheres indicate EdU-positive nuclei identified using the Spot tool in the Imaris software. **d** Number of EdU(+) cells per 1000 µm^2^ (mean ± SE, *n* = 3 per group). The individual data points are represented by gray dots. There was no significant difference between wild-type and *Prox1* cKO mice (*p* = 0.067, Student’s *t*-test). **e**–**g’** Transverse (**e**–**g**) and longitudinal (**e’**–**g’**) sections of the taste buds of *Prox1* cKO mice 4 days after EdU injection are shown as examples of EdU analysis from days 4 to 28. Arrowheads indicate EdU(+) nuclei within the taste buds. **h**, **h’** Temporal changes in the mean number (± SE) of EdU(+) cells per taste bud on days 4, 8, 12, 16, 20, and 28 after EdU injection are shown. Bars indicate the mean ± SE. The individual data points are shown as black dots. In wild-type mice (**h**), the number of EdU(+) cells remained nearly constant from days 4 to 12 and decreased after day 12. In *Prox1* cKO mice (**h’**), the number of EdU(+) cells decreased rapidly from day 4, exhibiting a biphasic decline with a transition on day 12. Asterisks (*) indicate significant differences (*p* < 0.05; two-way ANOVA, post hoc Tukey’s HSD test). Bold brackets represent the earliest time intervals showing statistically significant differences compared to day 4, whereas light brackets represent the earliest corresponding intervals relative to day 12. The decay curve for wild-type mice was fitted to a decreasing phase after day 12. For *Prox1* cKO mice, two distinct curves were fitted separately for the periods before and after day 12, when a change in the decay rate was observed. The decay curves are indicated by dashed lines after day 12 (magenta) for wild-type mice and before (green) and after (blue) day 12 for *Prox1* cKO mice. The equation used for curve fitting is *y* = *m1* × e^−*m2x*^, where *m1* represents the estimated initial value at *x* = 0 and *m2* is the decay constant. The lifespan of the taste bud cells was calculated using the decay constant from the fitted curves as follows: lifespan = 1/*m2*. The lifespan of taste bud cells in *Prox1* cKO mice was shorter than that in wild-type mice. **i**, **i’** Schematic presentation of the decay curves and calculated lifetimes from them. The colors of the curves and numbers in graph (**i**, **i’**) correspond to those in panel (**h**, **h’**). The areas filled with each color represent the temporal changes in taste bud cell populations. For the *Prox1* cKO mice, two cell populations (short-lived green and long-lived blue) were assumed. **j**, **j’** An example of a taste bud with an ovoid space containing small nuclear fragments in the taste buds of *Prox1* cKO mice on day 8 after EdU injection. EdU signals were colocalized with nuclear fragments (**j’**). Hoechst signals (**j’**) are shown with the gamma value adjusted to enhance the visibility of small nuclear fragments. Scale bar: 20 µm

Prior to analyzing cell lifespan, we investigated cell proliferation in the taste epithelium, which provides new cells to the taste buds. EdU was administered intraperitoneally, and the CV epithelium was collected 2 h after injection. The number of EdU(+) cells was counted per unit area (200 µm × 100 µm) (Fig. 8a–d). Areas were selected to contain a similar number of taste buds (27.3 ± 4.3, mean ± SD, *n* = 12). The mean number of EdU(+) cells per 1000 µm^2^ was 4.3 ± 0.2 in wild-type mice and 5.1 ± 0.2 in *Prox1* cKO mice (mean ± SE; Fig. 8d). Although the number of EdU(+) cells tended to be higher in *Prox1* cKO mice than in wild-type mice, this difference did not reach statistical significance (*p* = 0.067, Student’s *t*-test).

Next, we assessed the average lifespan of taste bud cells using KCNQ1 as a marker, which labels Type I–III cells. Following intraperitoneal administration of EdU, the CV epithelium was collected on days 4, 8, 12, 16, 20, and 28 after administration (Fig. 8e–h’). The lifespan of taste bud cells was estimated based on the temporal changes in EdU(+) cells per taste bud in EdU pulse-chase experiments (Fig. 8h, h’).

In wild-type mice, the number of EdU(+) cells remained nearly constant from days 4 to 12 and decreased after day 12. These results indicate that EdU(+) cells within the KCNQ1(+) cell population reached a peak level by day 4 and that EdU-labeled cells rarely died before day 12. This also means that the number of EdU(+) cells on day 4 could be useful as a benchmark indicating the number of newly supplied cells to taste buds following a single EdU injection.

The decay curve was fitted to the decreasing phase from day 12 onward (*R*^2^ = 0.88). Based on the fitted curve equation, the half-life and the average lifetime from day 12 onward were estimated as 6.6 days and 9.6 days, respectively (Fig. 8h, i). These estimations did not include the plateau period of 8 days between days 4 to 12 and the initial 4-day period before the first measurement, during which no data were collected. Therefore, by adding the 8-day plateau period to the lifetime of 9.6 days obtained from the decay curve, the average lifespan of KCNQ1(+) cells can be estimated as at least 17.6 days, excluding the initial unmeasured 4-day period.

In contrast to the wild-type mice, the number of EdU(+) cells in *Prox1* cKO mice decreased rapidly from day 4, exhibiting a biphasic decline with a transition on day 12 (Fig. 8h’). To simplify the analysis, we adopted a two-phase model assuming two cell populations composed of short- and long-lived cells (Fig. 8i’) and fitted two distinct decay curves to the data: one for days 4–12 and another for days after day 12. The average lifetime derived from the fitted decay curve for days 4–12 (*R*^2^ = 0.84) was 2.5 days, whereas that for days after day 12 (*R*^2^ = 0.94) was 6.0 days. Excluding the initial 4-day period, in *Prox1* cKO mice, the average lifespan of KCNQ1(+) cells was estimated to be at least 2.5 days before day 12 and at least 14 days after day 12 (i.e., 6.0 days + the 8-day period). Notably, in this model, long-lived cells in *Prox1* cKO mice still had a shorter lifespan than those in wild-type mice.

No statistically significant differences were observed between *Prox1* cKO mice and wild-type mice in the number of EdU(+) cells at any time points after EdU administration (Fig. 8h, h’), whereas the number of EdU(+) cell on day 4, which represent a benchmark for newly supplied cells to the taste buds, tented to be slightly higher in *Prox1* cKO mice (1.2 ± 0.1, mean ± SE) than in wild-type mice (0.9 ± 0.1, *p* = 0.758).

During EdU experiments, we observed that a few taste bud in *Prox1* cKO mice on day 8 contained ovoid spaces with small nuclear fragments, which were not observed in wild-type mice (Fig. 8j, j’). The prominent EdU signals over the nuclear fragments within these spaces enabled us to identify their presence in taste buds. These spaces had an ovoid shape, similar to the dark voids described above. However, they contained multiple small nuclear fragments and exhibited background signals for nuclear staining.

### Elevated apoptosis in the taste buds of *Prox1 *cKO mice

**Fig. 9:**
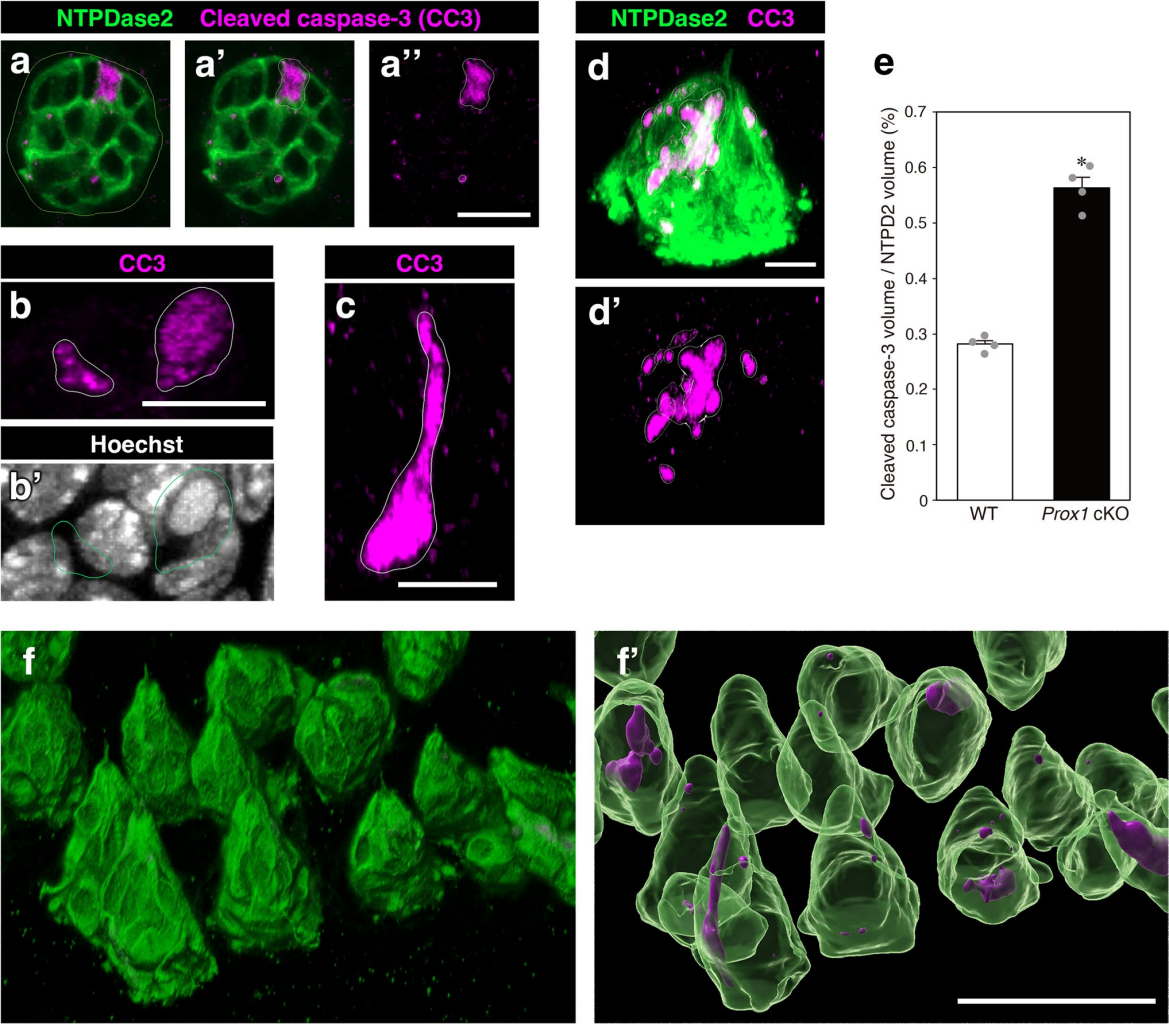
Quantification of apoptotic cell death in taste buds of the CV. Whole-mount immunohistochemistry was performed for NTPDase2 (green) and cleaved caspase-3 (CC3) (magenta). To measure the volume of CC3 signals and taste buds detected by NTPDase2 immunostaining, we generated surface objects that tightly enclosed the signal regions of taste buds and CC3 using the Surface tool in the Imaris software. **a**–**d’**, **f**, **f’** Example images obtained from *Prox1* cKO mice. **a**–**a”** Transverse optical section of the taste bud. The outlines of the taste bud (NTPDase2) surface object (**a**) and CC3 surface objects (**a’**, **a”**) are indicated by the lines. **b**, **b’** CC3 signals observed in the taste bud are shown together with nuclear staining (white; Hoechst) in transverse sections. The outlines of the CC3 surface objects are indicated by lines (**b**, **b’**). The right CC3 object includes an apoptotic body that is shown as a uniformly stained round-shaped nuclear staining (**b’**), but the left one does not. **c** An example of a CC3 signal showing taste cell-like morphology in the taste bud. The line indicates the outline of the surface object. **d**, **d’** 3D projection image of the taste bud. The outlines of the CC3 surface objects are indicated by the lines (**d**, **d’**). The CC3 signals were fragmented and scattered, and the cell-like morphology was not discernible. Most CC3 signals were difficult to assign to specific cellular or nuclear structures. **e** Summary of the percentage (%) of CC3 surface volume per taste bud (NTPDase2) surface volume (mean ± SE, *n* = 4 per group). The individual data points are represented by gray dots. The asterisk indicates a significant difference from that in wild-type mice (*p* < 0.05; Student’s *t*-test). The volume fraction of CC3 per taste bud in *Prox1* cKO mice was approximately twice that in wild-type mice. **f** 3D projection image of the taste buds at lower magnification. **f’** The corresponding surface objects generated from the 3D image shown in (**f**). NTPDase2 surface objects (green) are shown with CC3 objects (magenta) detected within them. Scale bars: 10 µm (**a**–**d’**) and 50 µm (**f**, **f'**)

The reduced size of taste buds and shortened lifespan of taste bud cells in *Prox1* cKO mice suggest an increase in cell death within taste buds. To quantify cell death, we performed whole-mount immunohistochemistry using CC3 as an apoptotic marker (Fig. 9). Previous studies that used CC3 as a marker quantified apoptosis by counting CC3(+) cells. However, in our whole-mount immunostaining, the CC3 signal patterns varied widely, ranging from cell-like shapes to scattered small particles (Fig. 9a–d’). Many CC3 signals were difficult to assign to specific cellular or nuclear structures. Therefore, we quantified apoptosis by calculating the ratio (%) of the volume of CC3 signals to the volume of taste buds detected using NTPDase2 as a marker. To reproducibly and consistently detect the boundaries of CC3 signals and taste bud profiles across whole-mount images using standardized criteria, we used the Surface tool in Imaris 10.0.2 (Fig. 9f, f’). The mean (± SE) percentage of CC3 signal volume per taste bud was 0.28 ± 0.01% in wild-type and 0.56 ± 0.01% in *Prox1* cKO mice. The fraction of CC3 signal volume per taste bud in *Prox1* cKO mice was approximately twice that of wild-type mice, and the difference between wild-type and *Prox1* cKO mice was statistically significant (*n* = 4 per group, *p* < 0.001; Student’s *t*-test) (Fig. 9e).

## Discussion

In the present study, we found that *Prox1* knockout reduced the number of taste bud cells by approximately half by affecting all Type I–III cell populations rather than specific cell types. In *Prox1* cKO mice, apoptosis was elevated, consistent with a shortened lifespan of taste bud cells. We also found that *Prox1* knockout increased the number of dark voids within the taste buds.

*Prox1* is a unique transcription factor in the taste buds. Its expression begins along with *Shh* in the taste primordia during the embryonic stage (Nakayama et al. 2015). Once taste buds are generated, *Shh* expression is limited to the basal Type IV cells of taste buds, but *Prox1* is expressed in all cell types (Type I–IV). During taste cell turnover, *Shh* is downregulated as cell differentiation progresses, but *Prox1* remains throughout the life of the taste bud cells. Therefore, the function of *Prox1* needs to be assessed by dissecting its function into two phases: embryonic and cell turnover. Our approach using *K5-Cre* to drive the conditional knockout of *Prox1* enabled us to assess its function during cell turnover.

### *Prox1* maintains the number of Type I–III cells

The structural and functional homeostasis of taste buds is maintained by various transcription factors, the expression patterns of which are formed according to their respective functions. The cell type-specific expression of these factors reflects their roles in the differentiation of specific cell types: Type II-specific *Pou2f3*, Type III-specific *Ascl1* and *Nkx2-2*, and sweet/umami and sodium-responsive cell-specific *Etv1*. Knockout of these factors results in the loss or decrease in a specific cell type or subpopulation of specific cell types in the taste buds (Matsumoto et al. 2011; Hsu et al. 2020; Qin et al. 2021; Ohmoto et al. 2023). In contrast, *Prox1* is expressed in all taste bud cells, regardless of cell type (Nakayama et al. 2015), and *Prox1* knockout in taste buds results in a reduction in all three cell types (Type I–III). The total number of cells decreased by approximately half. These results suggest that *Prox1* plays an essential role in the maintenance mechanism shared by all Type I–III cells during cell turnover in taste buds.

### Compensatory capacity maintaining taste bud size

The total number of cells per taste bud represents taste bud size and is determined by the balance between cell supply and taste cell lifespan, which depends on the frequency of apoptosis. Taste bud size would increase if cell supply were upregulated, and changes in lifespan could enhance or counteract this effect. Taste bud cells are postmitotic; cells generated around taste buds exit the cell cycle, enter the buds, and become *Shh*(+) precursor cells in the basal region (Miura et al. 2014; Finger and Barlow 2021)*.*

In terms of taste bud size maintenance, a previous study demonstrated a compensatory capacity of taste buds to maintain size and cell type composition constant against excess cell supply (Harrison et al. 2011). The *p27*^*kip1*^ gene encodes a cyclin-dependent kinase inhibitor, the downregulation of which promotes cell cycle exit in stem and progenitor cells (Besson et al. 2008). Knockout of this gene increases cell supply to the taste buds by promoting cell cycle exit (Harrison et al. 2011). However, in *p27*^*kip1*^ KO mice, neither the proportion of Type I–III cells nor the total cell number changed, unlike in other organs where hyperplasia and tissue disruption occurred (Kiyokawa et al. 1996; Nakayama et al. 1996). The equivalence between *p27*^*kip1*^ KO and wild-type taste buds was attributed to accelerated turnover, in which apoptosis increased and taste cell lifespan was shortened. These findings shed light on the compensatory capacity of taste buds through which the impact of increased cell supply is canceled by a shortened lifespan due to elevated apoptosis.

However, the molecular mechanisms governing taste cell lifespan remain unclear. In this study, we showed that *Prox1* knockout shortened the lifespan of taste bud cells, regardless of cell type, and upregulated apoptosis. These findings suggest that *Prox1* contributes to the molecular mechanisms that maintain taste bud size by regulating apoptosis and may preserve taste sensitivity by maintaining the number of sensory cells. Further investigation of *Prox1* in the taste buds of *p27*^*kip1*^ KO mice may deepen our understanding of the compensatory mechanisms underlying taste bud size homeostasis.

### Mediator of apoptosis in taste buds and potential involvement of ferroptosis

Among its various functions, *Prox1* has been reported to inhibit two different forms of programmed cell death: apoptosis and ferroptosis.

*Prox1* knockdown promotes apoptosis in oligodendrocyte precursor cells (OPCs) via selective upregulation of the BH3-only protein NOXA (Chang and Teng 2018). NOXA induces cytochrome c release and caspase activation via Bax and Bak activation (Croce et al. 2025). In the taste epithelium, fractionated head and neck irradiation increased NOXA expression and cell death detected by TUNEL in the CV and anterior tongue epithelium (Gaillard et al. 2019). Taken together, the elevated apoptosis in the taste buds of *Prox1* cKO mice may be mediated by NOXA.

In addition, *Prox1* restrains ferroptosis in colorectal carcinoma (Zhang et al. 2023). Although ferroptosis has not been reported in taste buds, it may contribute to the reduced cell numbers in *Prox1* cKO mice. Further investigation is required to assess the potential involvement of ferroptosis in this process.

Recent molecular approaches such as single-cell RNA sequencing enable analysis of cellular heterogeneity and molecular dynamics at the single-cell level in the taste buds (Vercauteren Drubbel and Beck 2023; Wang et al. 2025). By applying these techniques to *Prox1* cKO analysis, it will be possible to identify downstream targets of *Prox1* within taste buds and reveal the molecular cascades that regulate cell survival and death within taste buds.

### Dark voids and ovoid spaces containing small nuclear fragments

We identified two types of previously unreported ovoid spaces: dark voids lacking background signals of nuclear staining and ovoid spaces containing small nuclear fragments. Both are increased by *Prox1* knockout and reside in the upper half of the taste buds, where old or dying cells are located (Feng et al. 2014; Wilson et al. 2025), supporting the notion that both arise due to increased cell death within taste buds.

The latter, which contains nuclear fragments, appears to be associated with apoptosis. The small nuclear fragments are likely to be remnants of dead cells or apoptotic bodies. However, CC3 immunohistochemistry did not detect this structure in *Prox1* cKO mice, possibly because of the low frequency of these spaces. These spaces on day 8 after EdU injection suggest that some taste cells generated in the epithelium surrounding taste bud died within 8 days of their birth. Recently, many studies have reported that anticancer drugs or infection with bacteria and viruses increase cell death in taste buds (Cohn et al. 2010; Feng et al. 2014; Ren et al. 2023). However, this type of ovoid space where nuclear fragments accumulate has previously been unreported. It is likely that whole-mount immunohistochemistry visualizing the 3D structure of the taste bud employed in the present report enabled the identification of this space for the first time.

In contrast, dark voids are more unique. These voids increase with elevated apoptosis, but there is no indication of dead cell remnants inside, except in rare cases. We found dark voids by examining the faintly spreading background signal of nuclear staining, which is usually considered meaningless and overlooked. These voids were rare and small in wild-type mice, but *Prox1* knockout markedly enhanced both the frequency and size of the voids, making them discernible. Whole-mount 3D-based image analysis of taste buds provided morphological features: an ovoid shape embedded in the upper half of the taste buds. So far, they have only been detected as spaces lacking the background signals of nuclear staining. Further detailed characterization, such as using electron microscopy and immunohistochemistry, is necessary to clarify their precise characteristics. Nonetheless, dark voids appear to steadily reflect structural changes within taste buds caused by elevated apoptosis and may be related to a novel form of programmed cell death, such as erebosis (Ciesielski et al. 2022), which is characterized by the absence of nuclear signals, resulting in darkened spaces. Alternatively, they might represent the vacuolar degeneration of phagocytic cells within taste buds (Rezaie and Al-Sarraj 2007; Wilson et al. 2025).

### EdU labeling and cell supply to taste buds

We conducted birth dating and analyzed the average lifespan of taste bud cells using KCNQ1 as a marker. Although KCNQ1 is widely used as a taste bud cell marker, our unpublished data (manuscript in preparation) indicate that KCNQ1 is not expressed in immature taste bud cells. Therefore, birth dating using KCNQ1 as a marker started on day 4 after EdU injection, when KCNQ1 expression reliably marks maturing taste bud cells. Using this method in wild-type mice, the number of EdU(+) cells per taste bud was almost constant from days 4 to 12 and then decreased after day 12. The temporal changes in EdU(+) cells were similar to those reported in previous studies, which indicated that the decline in EdU(+) cells did not begin before day 10 (Perea-Martinez et al. 2013) and before day 14 (Horie et al. 2025) post-injection.

We reasoned that the stable phase from days 4 to 12 reflects the period during which all EdU-labeled cells supplied to the taste buds following a single EdU injection were fully incorporated and persisted within the taste bud without undergoing cell death. The number of EdU(+) cells observed on day 4 can be considered as a useful benchmark of the number of cells newly supplied to the taste buds following a single EdU injection.

In contrast to wild-type mice, EdU(+) cells were already in the reduction phase on day 4 post-injection in *Prox1 *cKO mice. There was a statistically significant difference in EdU(+) cell numbers between days 4 and 12. This suggests that the number of newly supplied cells may have been higher in *Prox1* cKO mice than in wild-type mice at earlier time points before day 4, although no statistically significant difference was observed in EdU(+) cell numbers between the two groups at day 4. If the cell supply to the taste buds increases in *Prox1* cKO mice, a compensatory mechanism could be involved in the taste buds, counterbalancing the decrease in mature cell types by increasing the cell supply. Further analysis of EdU(+) cells before day 4 is necessary to clarify the details of cell supply in *Prox1* cKO mice. However, the analysis using KCNQ1 is challenging because of the lack of KCNQ1 expression in immature cells. While PROX1 is a better marker expressed in all taste bud cells, it cannot be used to analyze *Prox1* cKO mice.

### Decay curve fitting and lifespan modeling

The number of EdU(+) cells in *Prox1* cKO mice exhibited a biphasic decline with a transition on day 12, whereas that in wild-type mice decreased in a monophasic manner after day 12. To simplify the model, we fitted two decay curves for *Prox1 *cKO mice before and after day 12 and a single curve for wild-type mice. The biphasic curves observed in *Prox1* cKO mice can be interpreted using the two-subpopulation model: one with a very short lifespan, which dies soon after differentiating into taste bud cells, and another with a longer lifespan, which survives through the period when most of the former population dies but still has a shorter lifespan than that in wild-type mice.

It is worth revisiting the study by Perea-Martinez et al., which revealed a similar temporal change in EdU(+) cells in wild-type mice using KCNQ1 immunohistochemistry (Perea-Martinez et al. 2013). They found that the decline in EdU(+) cells could be modeled not only by a single decay curve with a half-life of 11 days, but also by a combination of two decay curves with half-lives of 8 and > 24 days. Furthermore, they conducted birth dating using cell type-specific markers and demonstrated distinct half-lives among cell types: 8 days for Type II cells and 22 days for Type III cells, which closely matched the half-lives of 8 and 24 days predicted by birth dating using KCNQ1. Although the reduction of all three cell types in *Prox1* cKO mice likely supports the idea that the two-subpopulation model based on the two decay curves is applicable to all cell types (Type I–III), birth-dating analysis using cell-type-specific markers is needed to elucidate the details of the changes in the cell turnover of Type I–III cells caused by *Prox1* knockout.

## Conclusions

*Prox1* plays a critical role in various biological processes, including organ development and tissue homeostasis. Here, we reveal its novel function in taste buds. We demonstrate that *Prox1* is involved in the molecular mechanisms that govern the lifespan of taste bud cells, regardless of their cell type, during cell turnover. *Prox1* prevents apoptosis and prolongs the lifespan of taste cells. This may ensure taste sensitivity by maintaining the number of sensory cells within the taste buds. In addition, increased apoptosis leads to structural changes within the taste buds, which are detected by the appearance of dark voids. The analysis of such structural changes would provide valuable clues to understanding the clearance of dead cells within taste buds.

## Supplementary Information

Below is the link to the electronic supplementary material.

ESM 1 (XLSX 28.7 KB)

ESM 2 (TIF 916.2 MB)

ESM 3 (TIF 6.62 MB)

ESM 4 (TIF 83.2 MB)

ESM 5 (TIF 3.18 MB)

ESM 6 (DOCX 11.3 MB)

ESM 7 (MP4 46.8 MB 

## Data Availability

No datasets were generated or analysed during the current study.

## Abbreviations

Shh: Sonic hedgehog
KCNQ1: Potassium voltage-gated channel subfamily Q member 1
IP3R3: Inositol 1,4,5-trisphosphate receptor, type 3
CA4: Carbonic anhydrase 4
NTPDase2: Ectonucleoside triphosphate diphosphohydrolase 2
PLCβ2: Phospholipase Cβ2
SNAP25: Synaptosomal-associated protein 25 kDa
NOXA (PMAIP1): Phorbol-12-myristate-13-acetate-induced protein 1
Bad: Bcl-2-associated agonist of cell death
Bax: Bcl-2-associated X protein
Bak: Bcl-2 homologous antagonist/killer
TUNEL: Terminal deoxynucleotidyl transferase dUTP nick end labeling
