# Supplementary figures and images for: Prox1 maintains taste bud structure via inhibition of apoptosis
Source: Cell Tissue Res. 2026 Feb 5;403(2):16. doi: 10.1007/s00441-025-04040-7 (PMC12872690; doi:10.1007/s00441-025-04040-7)

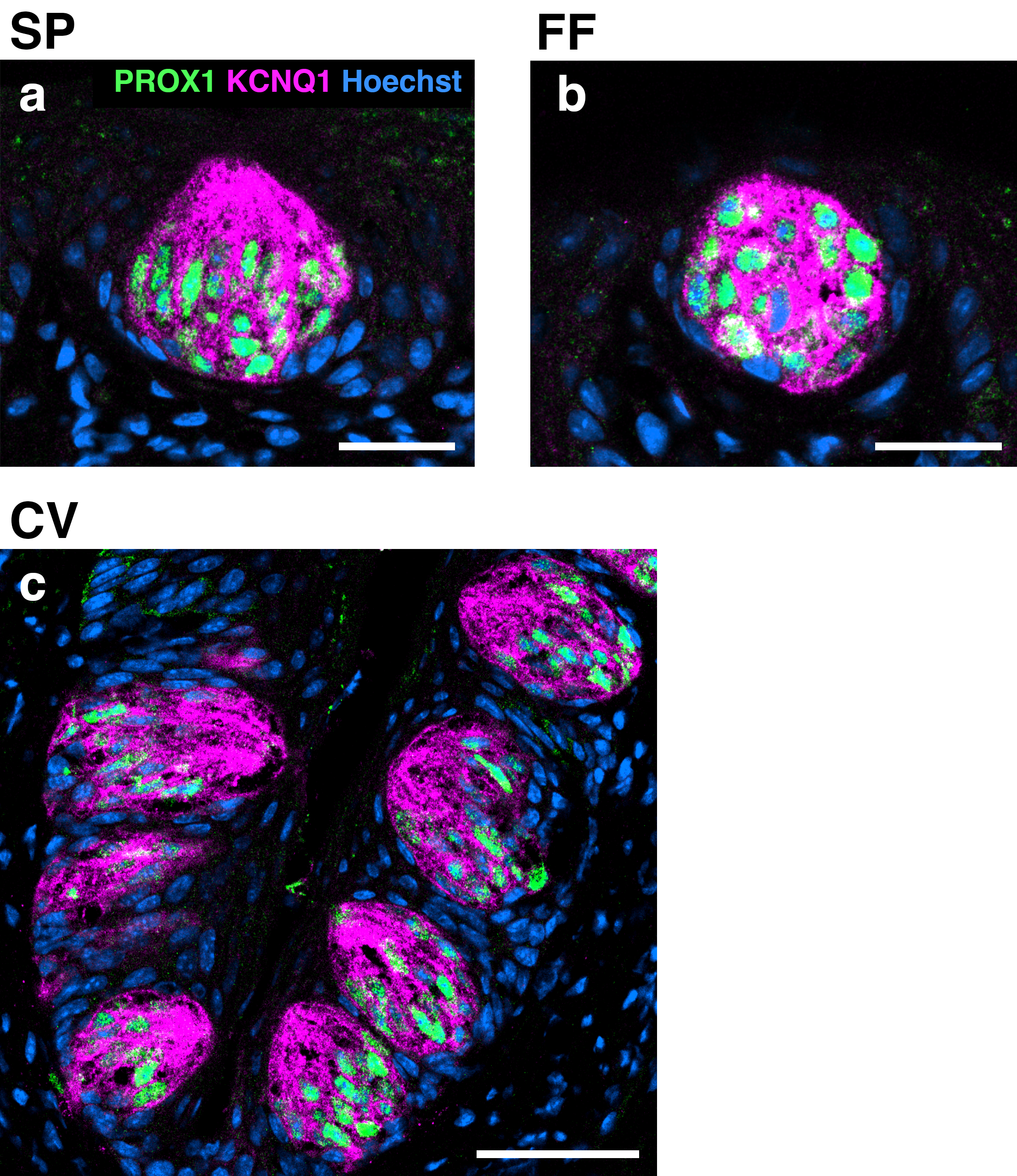

Supplement: Supplementary file 2 — (TIF 916.2 MB) [file 441_2025_4040_MOESM2_ESM.tif]

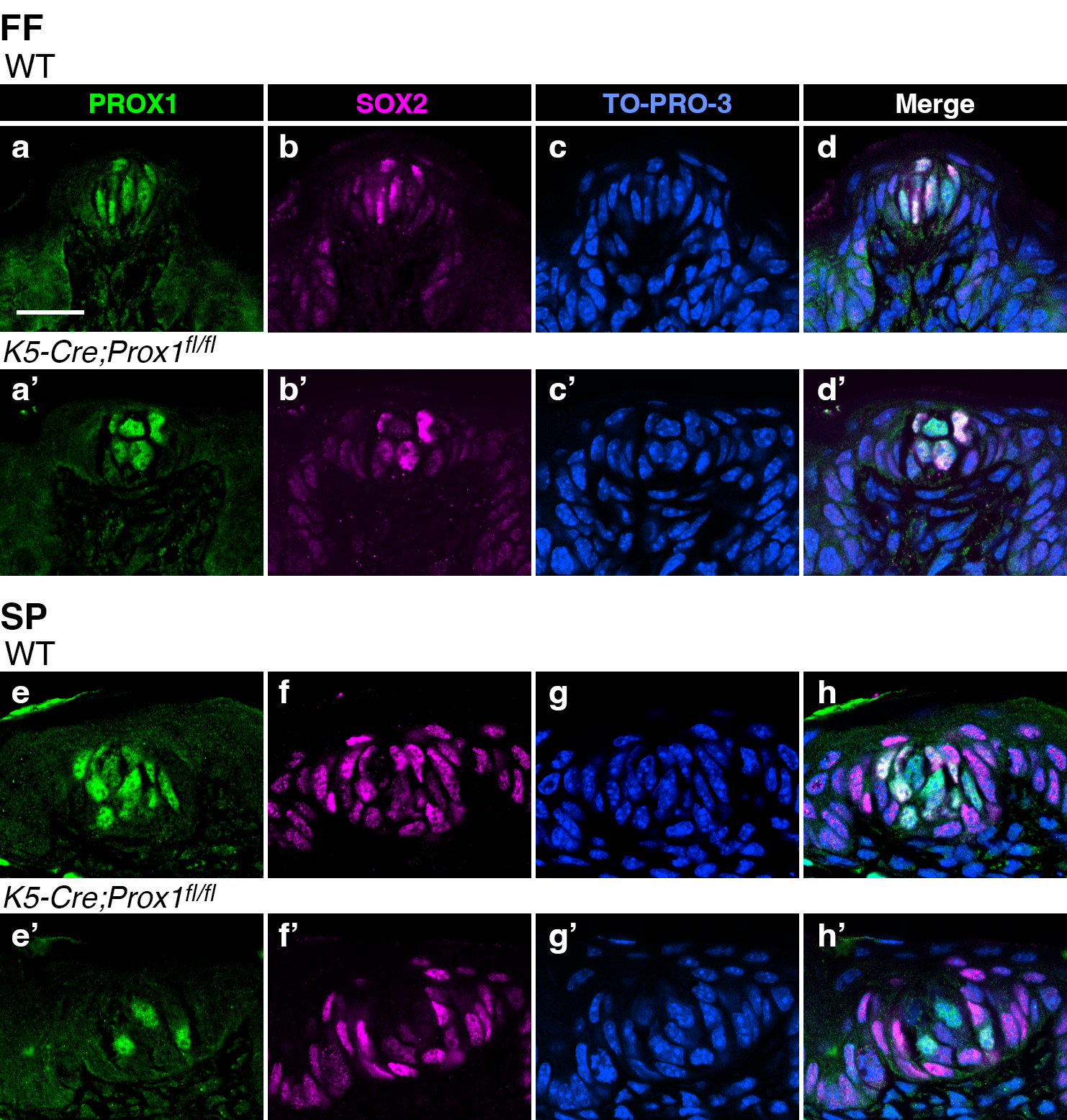

Supplement: Supplementary file 3 — (TIF 6.62 MB) [file 441_2025_4040_MOESM3_ESM.tif]

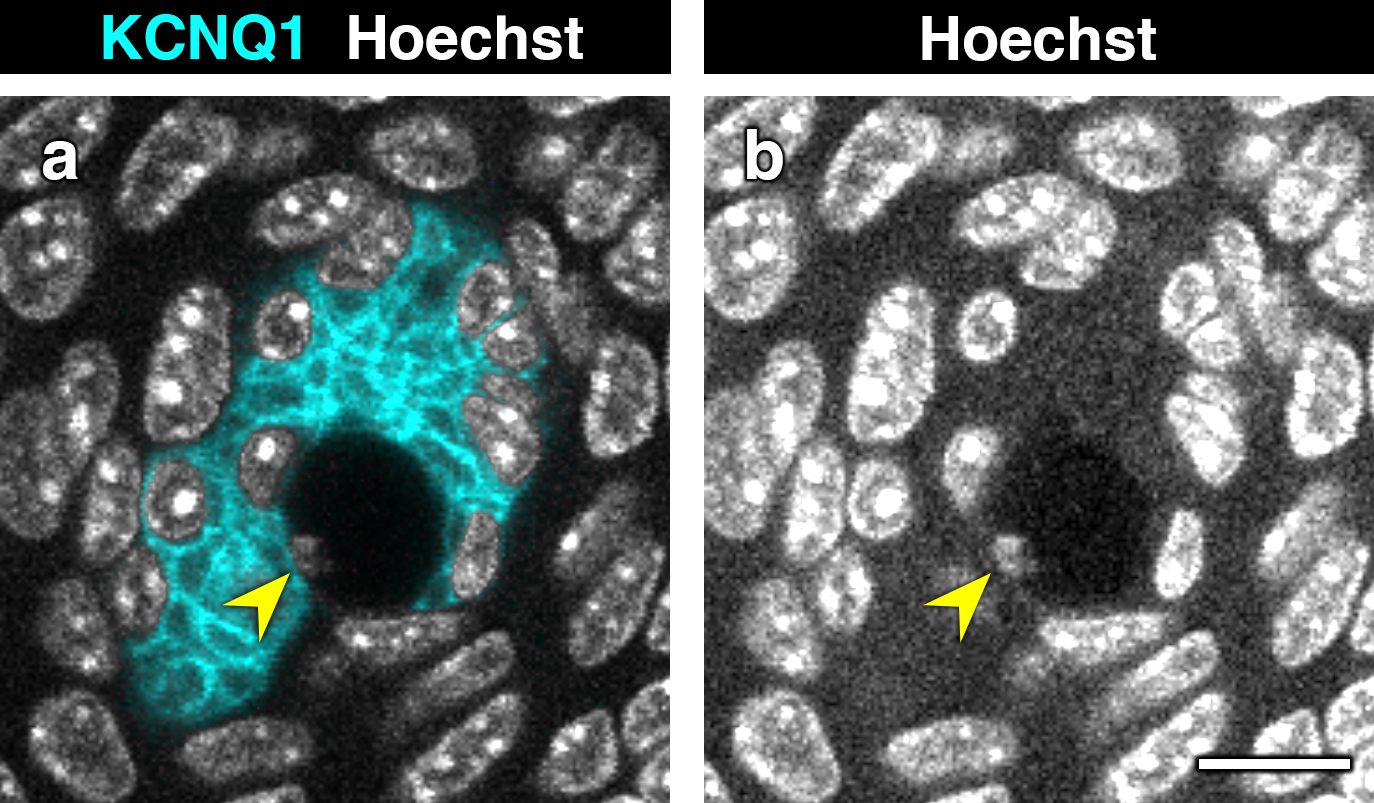

Supplement: Supplementary file 5 — (TIF 3.18 MB) [file 441_2025_4040_MOESM5_ESM.tif]
