# Supplementary material for: Prox1 maintains taste bud structure via inhibition of apoptosis
Source: Cell Tissue Res. 2026 Feb 5;403(2):16. doi: 10.1007/s00441-025-04040-7 (PMC12872690; doi:10.1007/s00441-025-04040-7)
Supplement: Supplementary file 6 — (DOCX 11.3 MB) [file 441_2025_4040_MOESM6_ESM.docx]

**Supplementary Table 1**Fixation conditions for whole-mount immunostaining with 4% PFA

| Primary antibody | Temperature | Time |
| --- | --- | --- |
| KCNQ1 | Room Temperature (RT) | 30 min |
| NTPDase2 | RT | 1 hour |
| PLCβ2 |  |  |
| SNAP25 |  |  |
| Cleaved caspase-3 |  |  |
| IP3R3 | RT | 15 min |
| CA4 |  |  |
| SHH | 4 ˚C | 1 hour |

**Supplementary Table 2**　Primary antibodies

| Target | Host | Dilution | Manufacturer | Cat. No. | RRID | Lot |
| --- | --- | --- | --- | --- | --- | --- |
| NTPDase2 | Sheep | 1/200 | R&D SYSTEMS (Minneapolis, MN) | AF5797 | AB_10572702 | CDUW01 10021 |
| PLCβ2 | Rabbit | 1/200 | SANTA CRUZ (Dallas, TX) | sc-206 | AB_632197 | A2908 |
| SNAP25 | Mouse | 1/200 | SIGMA-ALDRICH (Burlington, MA) | S5187-200UG | AB_261506 | 027K0990 |
| CA4 | Goat | 1/50 | R&D SYSTEMS (Minneapolis, MN) | AF2414 | AB_2070332 | WKG0111071 |
| KCNQ1 | Rabbit | 1/200 | MiLLIPORE (Temecula, CA) | AB5932 | AB_92147 | 4035062 |
| Cleaved caspase-3 | Rabbit | 1/100 | Cell Signaling Technology  (Danvers, MA) | 9661S | AB_2341188 | 45 |
| SHH | Goat | 1/100 | R&D SYSTEMS (Minneapolis, MN) | AF464 | AB_355373 | BIP1222071 |
| IP3R3 | Mouse | 1/50 | BD Biosciences (San Diego, CA) | 610313 | AB_397705 | 7173977 |
| PROX1 | Goat | 1/100 | R&D SYSTEMS (Minneapolis, MN) | AF2727 | AB_2170716 | VIY0113031 |
|  | Rabbit | 1/200 | MiLLIPORE (Temecula, CA) | AB5475 | AB_177485 | NG1734879 |
| SOX2 | goat | 1.6/600 | SANTA CRUZ (Dallas, TX) | sc-17320 | AB_2286684 | F0210 |

**Supplementary Table 3**Secondary antibodies

| Target | Host | Dilution | Manufacturer | Cat. No. | RRID | Lot | Conjugated dye |
| --- | --- | --- | --- | --- | --- | --- | --- |
| Sheep IgG | Donkey | 1/400 | Invitrogen (Carlsbad, CA) | A11015 | AB_141362 | 2785566 | Alexa Fluor 488 |
| Rabbit IgG | Donkey | 1/400 | Invitrogen (Carlsbad, CA) | A10040 | AB_2534016 | 1218269 | Alexa Aluor 546 |
| Rabbit IgG | Donkey | 1/400 | Invitrogen (Carlsbad, CA) | A31573 | AB_2536183 | 2359136 | Alexa Fluor 647 |
| Mouse IgG | Donkey | 1/400 | Jackson Immuno Research  (West Grove, PA) | 715-175-150 | AB_2340819 | 164092 | Cy5 |
| Mouse IgG | Donkey | 1/400 | Invitrogen (Carlsbad, CA) | A31570 | AB_2536180 | 412442 | Alexa Fluor 555 |
| Goat IgG | Donkey | 1/400 | Invitrogen (Carlsbad, CA) | A21082 | AB_10562400 | 501396 | Alexa Fluor 633 |
| Goat IgG | Donkey | 1/400 | Invitrogen (Carlsbad, CA) | A11055 | AB_2534102 | 34691A | Alexa Fluor 488 |


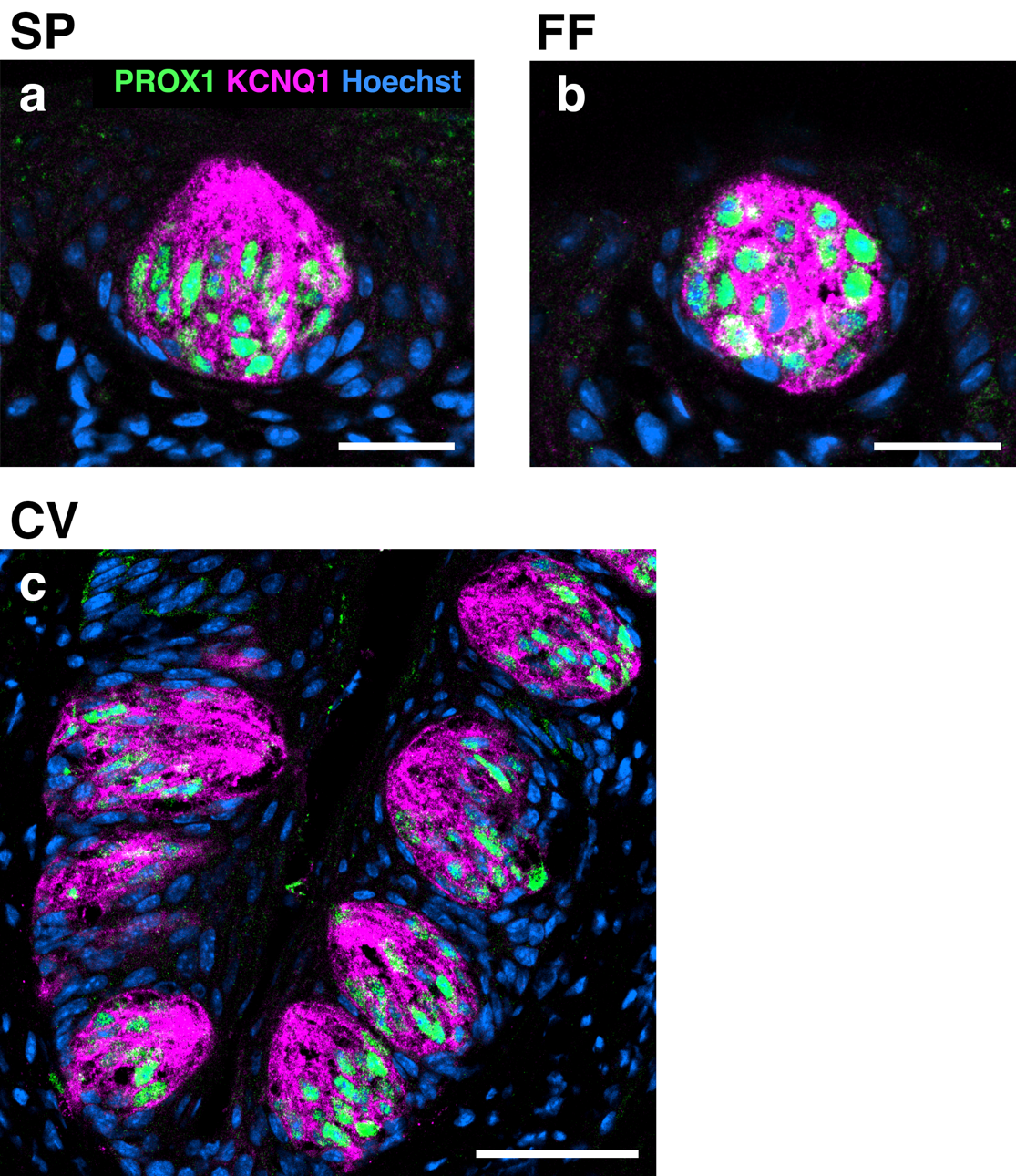


**Supplementary Figure 1** Immunohistochemistry of tissue sections from *Prox1^fl/fl^* mice showing PROX1 (green) and KCNQ1 (magenta) in the soft palate (SP; **a**), fungiform papillae (FF; **b**), and circumvallate papillae (CV; **c**). Scale bars: 20 µm (SP and FF) and 50 µm (CV)


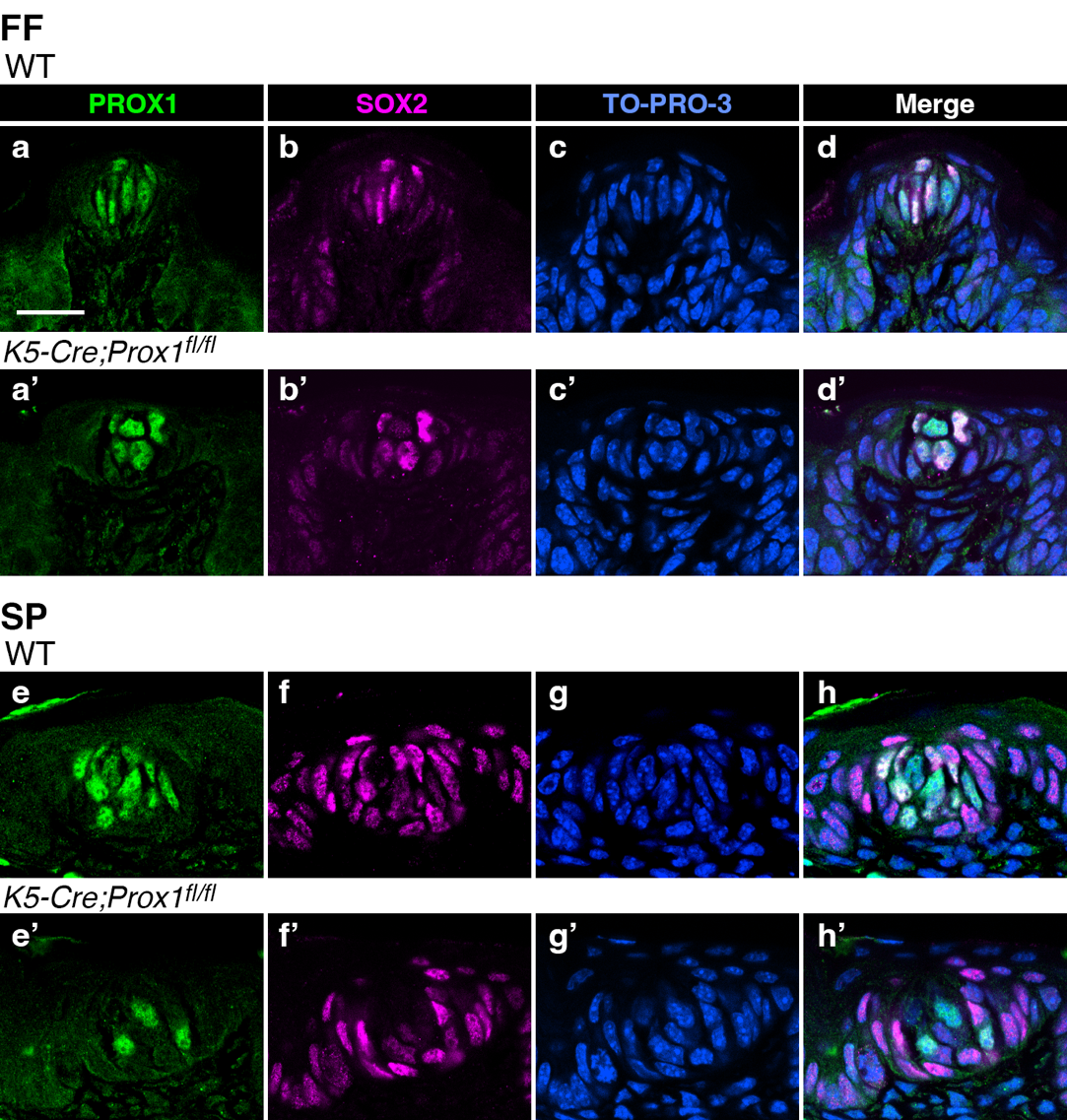


**Supplementary Figure 2** Immunohistochemistry on tissue sections showing PROX1 (green) and SOX2 (magenta) in the FF and SP of 2.5-day-old mice. In the FF, PROX1 expression was detected in *K5-Cre;Prox1^fl/fl^* mice (**a’-d’**) similar to that in wild-type mice (**a-d**). The intense expression of SOX2 overlapped PROX1 expression at 2.5 days after birth (Nakayama et al. 2015). In the SP of *K5-Cre;Prox1 ^fl/fl^* mice, most PROX1 was eliminated; however, a few PROX1(+) cells were occasionally detected in the taste buds (**e’-h’**). Scale bar: 20 µm


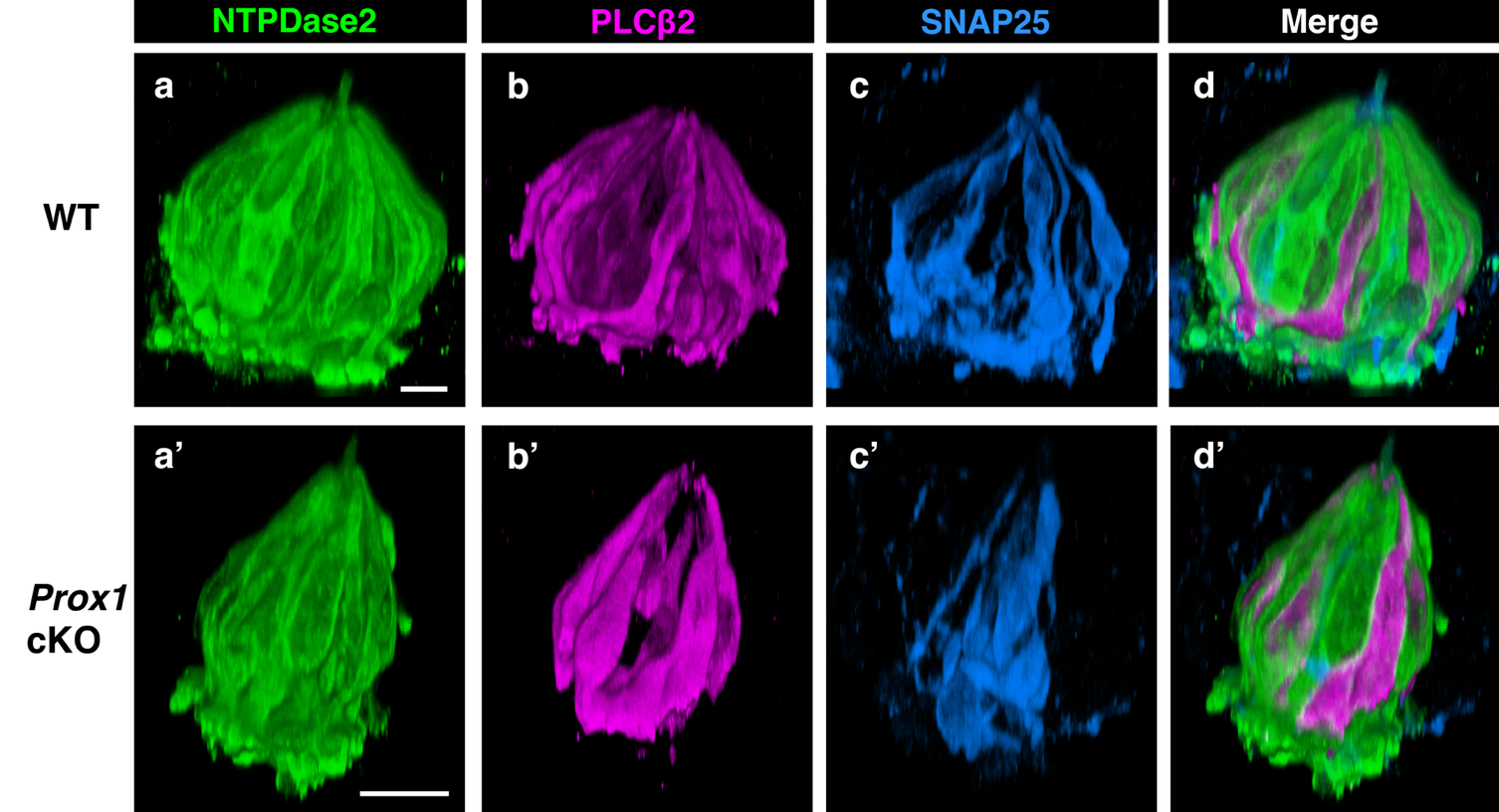


**Supplementary Figure 3** Triple-color whole-mount immunohistochemistry for Type I-III cell markers in example taste buds on the SP. The expression of NTPDase2 (green), PLCβ2 (magenta), and SNAP25 (blue) are shown. **a-d’** Three-dimensional (3D) images obtained using the blend mode in Imaris software from wild-type mice (**a-d**) and *Prox1* cKO mice (**a’-d’**). The taste bud of *Prox1* cKO mice was reduced in size. Scale bars: 10 µm


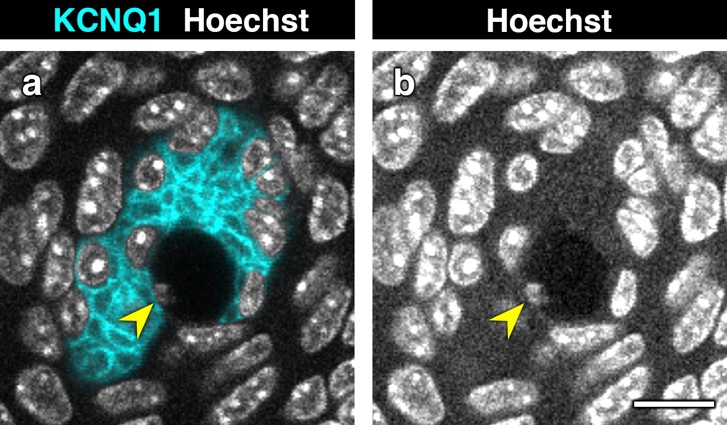


**Supplementary Figure 4** Dark void containing small nuclear fragments at the periphery. Whole-mount analysis. A transverse optical section is shown as an example of a taste bud with a dark void containing nuclear fragments in *Prox1* cKO mice. **b** Hoechst signal is shown with adjusted gamma values to enhance the visibility of the dark void. The Arrowhead indicates small nuclear fragments resembling apoptotic bodies inside the dark void. Scale bar: 10 µm
